# Global prevalence and epidemiological dynamics of bovine ephemeral fever: a misclassification-adjusted bayesian meta-analysis

**DOI:** 10.1186/s12917-026-05529-1

**Published:** 2026-05-04

**Authors:** Mobina Payami, Mohammad Erfan Aghighi, Narges Safari, Tania Akbari, Arghavan Sayahi, Parisa Izadkhah Shourehdeli, Mobina Pato, Arman Abdous, Farzane Shams

**Affiliations:** 1 Faculty of Veterinary Medicine, Karaj Branch, Islamic Azad University, Alborz, Iran; 2 Department of Clinical Sciences, Faculty of Veterinary Medicine, Karaj Branch, Islamic Azad University, Alborz, Iran; 3 Department of Veterinary Medicine, Garmsar Branch, Islamic Azad University, Semnan, Iran; 4 School of Pharmaceutical Sciences, Islamic Azad University, Tehran, Iran; 5 Department of Clinical Sciences, Faculty of Veterinary Medicine, SR.C, Islamic Azad University, Tehran, Iran; 6 Faculty of Veterinary Medicine, SR.C, Islamic Azad University, Tehran, Iran; 7 School of Public Health, Tehran University of Medical Sciences, Tehran, Iran; 8 Division of Neurological Sciences, Vetsuisse Faculty, University of Bern, Bern, Switzerland

**Keywords:** Bovine ephemeral fever, Seroprevalence, Bayesian hierarchical model, Meta-analysis, Cattle health

## Abstract

**Background:**

Bovine ephemeral fever (BEF) is an economically important yet under-characterized arboviral disease of cattle and water buffalo, with substantial impacts on productivity and herd health across tropical and subtropical regions. Despite decades of surveillance, global prevalence estimates remain uncertain due to pronounced diagnostic heterogeneity and uneven geographic coverage.

**Methods:**

We conducted a systematic review and Bayesian hierarchical meta-analysis of BEF serological and molecular prevalence data. Fifty-two studies published between 1967 and 2025 from 19 countries were included in the systematic review, of which laboratory-confirmed data from 16 countries contributed to quantitative meta-analysis and Bayesian modelling, representing 335,754 bovines. Observed prevalence estimates were corrected for imperfect diagnostic sensitivity and specificity using a misclassification-adjusted Bayesian framework, which improves accuracy by accounting for false positives and false negatives and harmonizing prevalence estimates across diagnostic methods, including differences between serological assays that measure cumulative exposure and molecular assays that detect short-lived active infection, and spatiotemporal models were applied to reconstruct national trends.

**Results:**

After correcting for diagnostic misclassification, the global true seroprevalence of BEF was estimated at 0.33. The corresponding misclassification-adjusted prevalence of active infection was 0.241; however, hierarchical modelling of outbreak-concentrated PCR and virus-isolation data produced a higher posterior mean of 0.56, reflecting the clustering of molecular sampling during epidemic periods. Marked geographic heterogeneity was observed, with posterior seroprevalence approaching 0.84 in Pakistan and Turkey, compared with substantially lower inferred immunity in Israel and Australia. Spatiotemporal analyses revealed declining long-term exposure in China and Australia, whereas recurrent outbreak-driven transmission persisted in parts of the Middle East and South Asia. Shifts in diagnostic practices over time, together with ongoing viral lineage diversification, further contributed to regional variation in reported prevalence.

**Conclusions:**

This study provides the first global, misclassification-adjusted synthesis of BEF epidemiology. The findings highlight the importance of standardized diagnostic protocols, enhanced genomic surveillance, and regionally targeted control strategies, as well as providing a solid quantitative baseline for future epidemiological assessment and policy development aimed at reducing the global burden of BEF.

**Supplementary Information:**

The online version contains supplementary material available at 10.1186/s12917-026-05529-1.

## Introduction

Bovine ephemeral fever (BEF) is an economically significant arboviral disease of cattle and water buffalo, characterized by acute febrile illness, reduced milk yield, weight loss, and sharp but transient declines in productivity. Caused by a member of the genus *Ephemerovirus* (family *Rhabdoviridae*) and transmitted primarily by hematophagous insects, BEF occurs across tropical, subtropical, and temperate regions of Asia, the Middle East, Africa, and Australia [1, 2]. Although mortality is typically low, the high morbidity associated with BEF leads to substantial economic losses, particularly through reduced milk production, temporary infertility, decreased draft power, and production interruptions in dairy and beef systems during large outbreaks [1, 3]. Expanding vector habitats and increasing climatic variability further accentuate the need for a clearer understanding of BEF transmission dynamics and global infection burden [1].

Despite decades of investigation, BEF epidemiology remains incompletely defined. Reported prevalence estimates vary widely within and between countries due to biological heterogeneity as well as inconsistent surveillance intensity, sampling designs, host populations, and diagnostic methods [4, 5]. Serological studies range from near-zero exposure in parts of Israel and Saudi Arabia to > 60–70% in outbreak-associated surveys in China, Turkey, and Pakistan, while molecular detection spans from negligible PCR positivity during inter-epidemic periods to > 90% during acute outbreaks in Taiwan, Iran, and Turkey [4, 6, 7]. Such variability complicates inference on transmission intensity, identification of high-risk regions, and reconstruction of long-term epidemiological trends. Temporal fragmentation further limits comparability, with many countries represented by only a few studies spread over several decades [2].

Interpretation of BEF prevalence data is further challenged by imperfect and evolving diagnostic assays. Early surveillance relied on virus neutralization tests (VNT) and virus isolation methods that are labor-intensive, variable in sensitivity, and prone to false negatives [8]. From the 1990s onwards, ELISAs and PCR assays provided improved analytical performance and greater standardization, but the coexistence of diagnostic eras introduces systematic misclassification: VNT underestimates seroprevalence relative to ELISA, and virus isolation underestimates active infection relative to PCR [9]. Importantly, serological assays (VNT and ELISA) measure cumulative antibody exposure, whereas PCR and virus isolation detect transient viremia, meaning that diagnostic choice can substantially influence observed prevalence independent of true transmission intensity. Conventional meta-analytic approaches that treat observed positives as true positives cannot correct for these biases and may distort global estimates or obscure genuine epidemiological patterns [10, 11].

Advanced statistical methods are needed to integrate heterogeneous data while adjusting for diagnostic error. Bayesian hierarchical modeling provides a principled framework for estimating true prevalence and propagating uncertainties arising from sampling variability, study heterogeneity, and assay misclassification [10, 12]. Treating diagnostic sensitivity and specificity as probabilistic quantities enables reconstruction of latent prevalence distributions, while hierarchical structures allow partial pooling across countries, species, and time periods [11, 12]. When combined with spatiotemporal regression, these models can recover long-term trends even under sparse or irregular sampling.

Despite BEF’s broad geographic distribution, no prior study has synthesized the complete global serological and molecular evidence base or systematically corrected for diagnostic misclassification when estimating true prevalence. Existing reviews are descriptive, regional, or outbreak-focused and do not integrate multiple diagnostic eras or evaluate heterogeneity, publication bias, or temporal trajectories [1]. The absence of a harmonized, methodologically robust global synthesis has hindered accurate assessment of BEF’s burden and its evolution through time.

To address these gaps, we conducted a comprehensive systematic review and meta-analysis encompassing serological and active-infection data from 52 studies published between 1967 and 2025 across 19 countries in the systematic review, with 16 countries contributing laboratory-confirmed data to quantitative modelling. We applied a misclassification-adjusted Bayesian framework to correct for imperfect diagnostic sensitivity and specificity and to enable direct comparison of prevalence estimates across assays, countries, and time periods. Our objectives were to: (1) estimate global and country-specific true seroprevalence and active-infection prevalence using misclassification-adjusted Bayesian hierarchical models; (2) quantify heterogeneity by diagnostic method, host species, and region; (3) reconstruct national temporal trends using spatiotemporal Bayesian regression; (4) characterize global transitions in diagnostic practice and their epidemiological implications; and (5) assess publication bias and methodological drivers of variation in reported prevalence. This approach provides a unified and diagnostically harmonized global synthesis of BEF epidemiology.

## Methods

### Literature search strategy

A systematic search was conducted to identify all studies reporting serological or molecular prevalence of Bovine Ephemeral Fever Virus (BEFV) in bovine species. Six databases (PubMed, Ovid MEDLINE, Ovid EMBASE, Scopus, Web of Science Core Collection, and CAB Abstracts) were searched using database-specific combinations of controlled vocabulary and free-text terms related to BEFV and epidemiological outcomes. These databases were selected because they collectively provide comprehensive coverage of veterinary medicine, infectious diseases, epidemiology, and zoonotic research, with CAB Abstracts offering specialized indexing of animal health and agricultural literature. Search strategies incorporated MeSH, Emtree, and CAB Thesaurus terms together with synonyms such as “Bovine ephemeral fever,” “BEFV,” “three-day sickness,” “ephemeral fever virus,” “seroprevalence,” and “epidemiology,” as detailed in Appendix 1. No restrictions were placed on language, publication year, or study design, and species filters were applied only when supported by the database to limit retrieval to animal studies. All searches were run on 8 December 2025, yielding 2,462 records across databases. Reference lists of all eligible articles were hand-searched to identify additional studies not captured electronically. The search and documentation procedures adhered to PRISMA guidelines to ensure completeness and reproducibility.

### Eligibility criteria

Studies were eligible for inclusion in the systematic review if they reported primary data on the occurrence or prevalence of Bovine Ephemeral Fever Virus (BEFV) infection in bovine species, specifically cattle (*Bos taurus*), water buffalo (*Bubalus bubalis*), or yak (*Bos grunniens*). Studies that included additional ruminant species, such as goats, sheep, or wildlife, were retained only when bovine-specific data could be extracted. Eligible study designs comprised cross-sectional surveys, outbreak investigations, seroepidemiological studies, and molecular detection studies that provided the number of animals tested and the number positive, or sufficient information to derive these values.

Studies using laboratory confirmation methods, including virus neutralization tests (VNT), ELISA, PCR assays, or virus isolation, were considered eligible for quantitative synthesis. Investigations relying exclusively on clinical diagnosis without laboratory confirmation were retained for descriptive purposes in the systematic review but were excluded from quantitative prevalence estimation and meta-analytic modelling, owing to the inability to account for diagnostic misclassification in such data.

Studies were excluded entirely if they lacked extractable prevalence data, used diagnostic approaches unsuitable for standardized interpretation, or did not allow separation of bovine-specific results. Experimental challenge studies, vaccine trials without natural infection data, narrative reviews, and duplicated datasets were removed, retaining only the most complete version when overlapping populations were identified. Full texts that could not be retrieved after repeated attempts were also excluded. These criteria ensured that the quantitative analyses were based on methodologically robust and diagnostically interpretable estimates of BEFV prevalence, while the systematic review remained comprehensive. In total, 52 studies representing 19 countries were included in the systematic review. Of these, studies providing laboratory-confirmed data (PCR, ELISA, VNT, or virus isolation) from 16 countries were eligible for quantitative meta-analysis and Bayesian modelling. Studies from Bangladesh, Nepal, and Japan relied exclusively on clinical diagnosis and were therefore retained for descriptive completeness but excluded from quantitative synthesis.

### Study selection

**Fig. 1:**
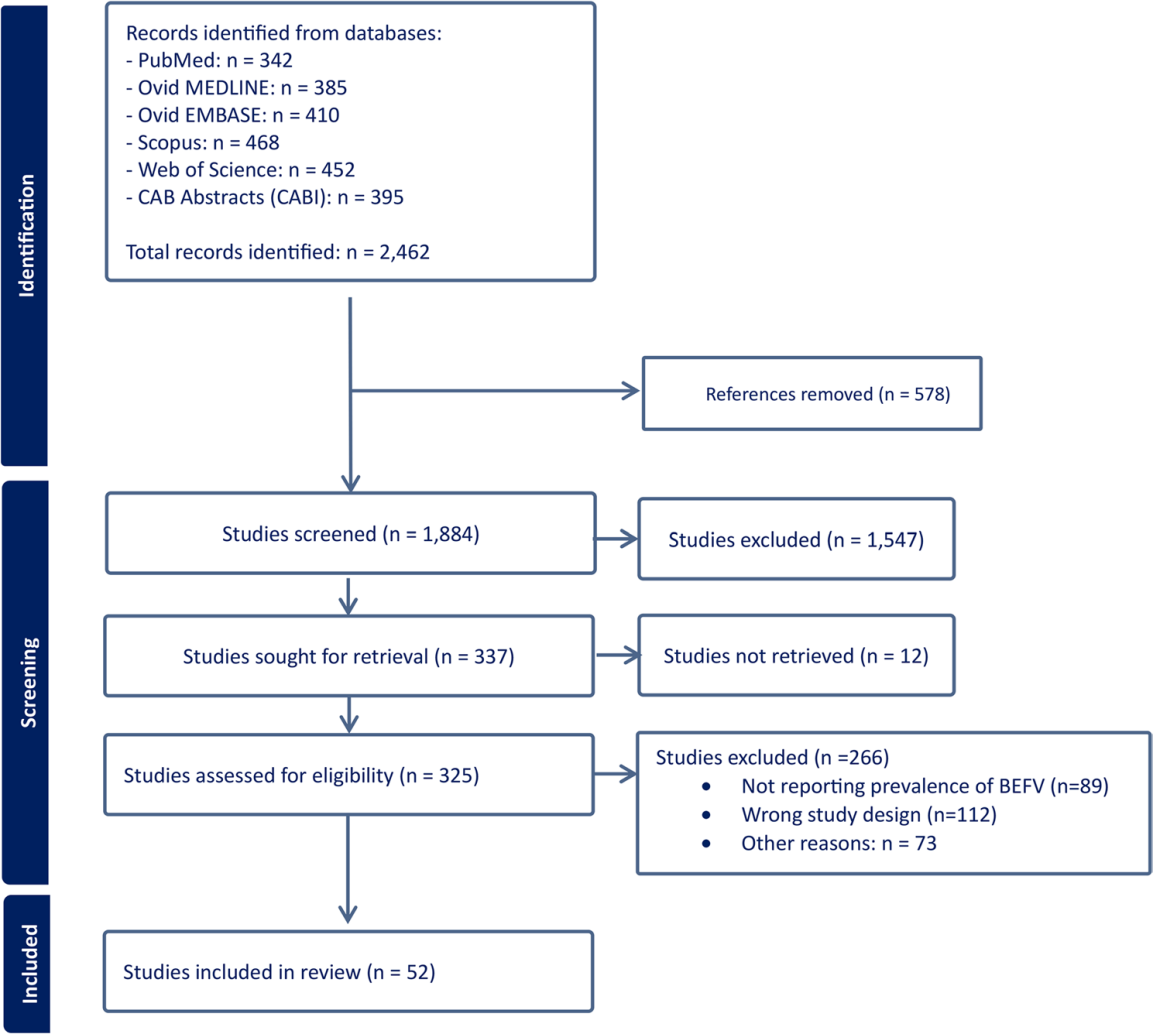
PRISMA flow chart of study selection

All retrieved records were imported into Covidence (Veritas Health Innovation, Melbourne, Australia), a web-based platform for systematic review management, and were automatically and manually deduplicated prior to screening. Study selection followed a two-stage process in accordance with PRISMA guidelines (Fig. 1). Titles and abstracts were first screened to identify studies reporting BEFV occurrences or prevalences in bovine species. Records clearly lacking epidemiological relevance were excluded at this stage. Full texts were obtained for all studies meeting the initial screening criteria or requiring further clarification. Of 1,884 screened records, 337 underwent full-text review, and 325 were successfully retrieved. Studies were excluded at this stage if they lacked extractable prevalence data, employed non-standard or uninterpretable diagnostic approaches, or used study designs incompatible with prevalence estimation. Twelve articles could not be retrieved despite repeated attempts and were excluded. Ultimately, 52 studies representing 19 countries met the criteria for inclusion in the systematic review. Among these, studies with laboratory-confirmed outcomes (PCR, ELISA, VNT, or virus isolation) were included in the quantitative meta-analysis and Bayesian modelling, whereas studies based solely on clinical diagnosis were summarized descriptively and excluded from quantitative synthesis.

### Data extraction

Data were extracted from all eligible studies using a standardized template to ensure consistency. For each study, we recorded the first author, publication year, country, sampling period, and host species, including cattle, water buffalo, yak, and other ruminants when applicable. Diagnostic methods were coded as virus neutralization test (VNT), ELISA, PCR, or virus isolation. For quantitative synthesis, each unique combination of study, diagnostic assay, host species, and sampling period was treated as an independent prevalence stratum to account for methodological heterogeneity. When multiple assays, species-specific results, or sampling periods were reported, data were abstracted separately at the finest level compatible with consistent synthesis while retaining publication-level identification and avoiding duplication of individuals. The numbers of animals tested and positive were extracted directly or derived from reported percentages. When available, subgroup information such as breed, sex, production type, vaccination status, outbreak context, and ecological descriptors was also recorded to support stratified analyses. All extracted variables were checked for internal consistency and completeness prior to meta-analysis and Bayesian modelling, and publication-level characteristics are summarized in Table 1.

**Table 1:** Characteristics of studies included in the systematic review of bovine ephemeral fever virus (BEFV) prevalence

#	First Author, Year	Country	Sampling Period	Pooled Diagnostic Methods	Species Included	Total Tested	Total Positive	Publication Included in Quantitative Meta-analysis
1	Cybinski, 1983 [13]	Australia	1972–1980	VNT	Buffalo, Sheep, Goat	334	14	Yes
2	St. George, 1985 [8]	Australia	1981–1984	Virus Isolation	Cattle	9	5	Yes
3	Uren, 1987 [2]	Australia	1983–1985	VNT, Clinical Symptoms	Cattle	2,321	888	Yes
4	Finlaison,2010 [14]	Australia	2008	VNT, Virus Isolation	Not specified	140	30	Yes
5	Finlaison, 2014 [7]	Australia	2009–2010	PCR, Virus Isolation	Not specified	330	139	Yes
6	Islam, 2018 [15]	Bangladesh	2017–2018	Clinical Symptoms	Crossbred cattle	1,050	55	No
7	Wenbin, 1991 [16]	China	1971–1987	VNT	Chinese Yellow	21,000	8500	Yes
					Friesian	1457	987	Yes
8	Li, 2015 [4]	China	2012–2014	ELISA	Cattle, Dairy, Buffalo, Yak	2822	899	Yes
9	Liu, 2016 [17]	China	2012–2015	ELISA	Yak	1,123	454	Yes
10	Zheng, 2015 [18]	China	2013	Virus Isolation	Cattle	21	9	Yes
11	Kun,2020 [19]	China	2013–2017	PCR	Cattle	9	5	Yes
12	El-Allawy, 2021 [20]	Egypt	2018–2019	ELISA, PCR	Buffalo, Cattle	134	48	Yes
13	Mohapatra, 2022 [21]	India	2018	ELISA	Cattle, Buffalo	92	27	Yes
14	Bazargani, 2013 [6]	Iran	2006	Clinical Symptoms	Cattle	2,097	274	No
15	Mirzaie, 2017 [22]	Iran	2013	PCR	Cattle	25,000	1,800	Yes
16	Rezatofighi,2022 [23]	Iran	2018,2020	VNT	Cattle	90	90	Yes
17	Yeruham, 2007 [24]	Israel	1991	Clinical Symptoms	Cattle	3,680	96	No
18	Yeruham, 2003 [25]	Israel	1999	VNT	Cattle	320	126	Yes
				Clinical Symptoms		4110	1586	
19	Yeruham, 2008 [26]	Israel	1999–2001	VNT	Cattle	400	233	Yes
				Clinical Symptoms		5081	2886	
20	Aziz-Boaron,2012 [27]	Israel	2006–2008	Clinical Symptoms	Cattle	948	7	No
21	Aziz-Boaron, 2013 [5]	Israel	2000–2009	VNT	Buffalo, Wildlife (7 spp.)	942	9	Yes
22	Behar, 2022 [28]	Israel	2015–2020	VNT	Cattle	234	11	Yes
23	Lavon, 2023 [3]	Israel	2021	PCR	Cattle	13,398	5,371	Yes
24	Golender, 2024 [29]	Israel	2023	PCR	Cattle	1,176	410	Yes
25	Al-Sultany, 2013 [30]	Iraq	2012	PCR	Cross-bred cattle	150	37	Yes
26	Hijazeen, 2020 [31]	Jordan	2012	VNT	Cattle	300	136	Yes
27	Ogawa, 1992 [32]	Japan	1988	Clinical Symptoms	Cattle	13,100	1,005	No
28	Sah, J ,2002 [33]	Nepal	1998–2000	Clinical Symptoms	Cattle	63	63	No
29	Zahid, 2018 [34]	Pakistan	2014	PCR	Cattle, Buffalo	600	340	Yes
30	Abdullah, 2020 [35]	Pakistan	2014	PCR	Cattle	50	33	Yes
31	Nadeem, 2024 [36]	Pakistan	2022	VNT	Buffalo, Cattle	300	126	Yes
32	Farag, 1998 [37]	Saudi Arabia	1990, 1996	VNT	Cattle	168	105	Yes
33	Abu-Elzein, 2006 [38]	Saudi Arabia	1993–1995	Clinical Symptoms, VNT	Cattle	977	0	Yes
34	Abu Elzein, 1999 [39]	Saudi Arabia	1996	VNT	Cattle	50	0	Yes
35	Zaghawa, 2017 [40]	Saudi Arabia	2007–2011	Virus Isolation	Cattle	4,800	1,601	Yes
36	Zaghawa, 2016 [41]	Saudi Arabia	2010	VNT	Dairy & Beef cattle	1480	276	Yes
37	Hwang, 2021 [42]	South Korea	2003–2011	VNT	Cattle, Sheep, Goat	3840	458	Yes
38	Kim, 2015 [43]	South Korea	2010	VNT	Cattle	500	14	Yes
39	Grobler, 2025 [9]	South Africa	2020–2022	Virus Isolation	Cattle	88	21	Yes
40	Chaisirirat, 2018 [44]	Thailand	2013–2017	PCR	Cattle	75	24	Yes
41	Wang, 2001 [45]	Taiwan	1967–1999	Clinical Symptoms	Cattle	181,555	26,560	No
42	Liao, 1998 [46]	Taiwan	1996	Clinical Symptoms, Virus Isolation	Cattle	111,208	15,954	Yes
43	Hsieh, Y. C,2005 [47]	Taiwan	2001–2002	Clinical Symptoms	Cattle	26,292	2,046	No
44	Tonbak, 2013 [48]	Turkey	2012	PCR	Cattle	60	48	Yes
45	Alkan, 2017 [49]	Turkey	2012	PCR	Cattle	27	13	Yes
46	Dik, 2014 [50]	Turkey	2012	PCR	Cattle	35	13	Yes
47	Tokgoz, 2023 [51]	Turkey	2020	ELISA, PCR	3 breeds (Holstein, Simmental, Brown Swiss)	249	168	Yes
48	Karayel-Hacioglu,2021 [52]	Turkey	2020	Virus Isolation	Cattle	22	22	Yes
49	Abayli, 2023 [53]	Turkey	2020	PCR	Cattle	32	23	Yes
50	Paksoy, 2024 [54]	Turkey	2020	PCR	Cattle	40	21	Yes
51	Özyörük, 2025 [55]	Turkey	2020	Clinical Symptoms, PCR	Simmental, Holstein Friesian	8107	2980	Yes
52	Anderson, 1998 [56]	Zimbabwe	1989–1995	VNT	Buffalo, 7 antelope spp.	1315	79	Yes

*Abbreviations*: *BEFV* Bovine ephemeral fever virus, *PCR* Polymerase chain reaction, *ELISA* Enzyme-linked immunosorbent assay, *VNT* Virus neutralization test, *spp*. *species*. When studies reported multiple diagnostic assays, species-specific results, or analytically distinct sampling components, data were extracted as separate prevalence strata for quantitative synthesis. The inclusion column reflects publication-level eligibility, whereas statistical analyses were conducted at the assay–stratum level

### Study quality assessment

The methodological quality of included studies was assessed using a modified Newcastle–Ottawa–style framework specifically adapted for cross-sectional serological and molecular prevalence surveys. The adapted tool evaluated three domains: selection, case ascertainment, and outcome reporting using seven binary criteria relevant to BEFV epidemiology. These criteria were assessed: (1) representativeness of the sampling frame; (2) clarity of inclusion and exclusion criteria; (3) adequacy of reported sample size; (4) use of a validated diagnostic assay; (5) reporting of the diagnostic protocol and cut-off values; (6) clarity of the numerator and denominator used for prevalence estimation; and (7) reproducibility of outcome reporting.

Each criterion was scored as 0 or 1, yielding a total quality score ranging from 0 to 7. Studies scoring 6–7 were classified as *low risk of bias*, scores of 3–5 as *moderate risk*, and scores of 0–2 as *high risk of bias*. Quality ratings were used descriptively to evaluate methodological rigor across studies and did not influence inclusion in the meta-analysis. Common methodological limitations included outbreak-based sampling with limited population representativeness and incomplete reporting of diagnostic validation parameters, particularly sensitivity and specificity for older assays. This structured approach ensured transparent and systematic assessment of study quality across diverse diagnostic methods, sampling strategies, and reporting practices (Supplementary Table 1).

### Conventional meta-analysis

Observed prevalence estimates were synthesized using a random-effects meta-analytic framework to characterize empirical variation before misclassification adjustment. Prevalence proportions were transformed using a logit transformation to stabilize variances and improve the normality of sampling errors, and pooled estimates were subsequently back-transformed for interpretation. Between-study heterogeneity was quantified using Cochran’s Q statistic, the I² metric, and the between-study variance (τ²), allowing assessment of variability beyond sampling error, where a statistically significant Q and high I² values indicate that observed differences in prevalence reflect true between-study heterogeneity rather than random variation alone. Subgroup analyses were conducted by diagnostic method virus neutralization test, ELISA, PCR, and virus isolation, and by host species, including cattle, water buffalo, yak, goats, and sheep, to identify systematic differences attributable to assay type or host. Forest plots were inspected to evaluate the influence of individual studies and to visualize between-study variability. These analyses provided a descriptive foundation for the subsequent estimation of misclassification-adjusted and hierarchically pooled prevalence in the Bayesian framework.

### Meta-regression and risk factor analysis

Meta-regression was conducted to investigate sources of between-study heterogeneity in BEFV prevalence. Univariable and multivariable random-effects meta-regression models were fitted with logit-transformed prevalence as the outcome and diagnostic method, host species, and publication year as covariates. Regression coefficients were exponentiated to obtain prevalence ratios with 95% confidence intervals. In studies reporting sex-specific data, a random-effects model was used to estimate pooled odds ratios for seropositivity in males versus females, using study-specific log odds ratios and their standard errors. Heterogeneity in these models was summarized by τ² and I², and the statistical significance of the covariates was assessed using Wald tests.

### Publication bias assessment

Publication bias was assessed using both graphical and statistical methods. Funnel plots were examined to evaluate the symmetry between study precision and observed prevalence, with asymmetry interpreted in the context of the inherent heterogeneity of prevalence data. Egger’s regression test was used to detect small-study effects, and Begg’s rank correlation provided a complementary nonparametric assessment. When asymmetry was indicated, the trim-and-fill procedure was applied to estimate the number of potentially missing studies and to generate adjusted pooled prevalence values. These analyses were conducted separately for each diagnostic method to identify assay-specific patterns in reporting. Together, these evaluations enabled assessment of whether selective publication influenced the synthesized prevalence estimates.

### Misclassification-adjusted prevalence estimation

Observed BEFV prevalence values were first corrected for diagnostic misclassification using the Rogan–Gladen approach [10]. Sensitivity and specificity for each assay type were modelled as beta-distributed random variables with priors centred on assay-specific mean performance and parameterized to reflect realistic between-study variability, following established Bayesian misclassification frameworks [11, 12]. These diagnostic parameters were repeatedly sampled to propagate uncertainty, linking the observed proportion of positive animals to a posterior distribution of the underlying true prevalence in accordance with contemporary Bayesian latent-class modelling practice [57]. For each study, the resulting distribution of adjusted prevalences was summarized using the median and the 2.5th and 97.5th percentiles to form 95% simulation intervals (SI). This procedure produced misclassification-adjusted prevalence estimates that were internally coherent and directly comparable across diagnostic methods, host species, and countries, explicitly accounting for systematic differences between assays such as ELISA versus VNT for serology and PCR versus virus isolation for active infection, and served as the harmonized inputs for subsequent hierarchical modelling.

### Bayesian hierarchical modelling

The misclassification-adjusted prevalence distributions were then incorporated into a Bayesian hierarchical logistic model in which true prevalence was treated as a latent parameter composed of a global mean and random deviations at the country and species levels. Weakly informative normal priors were assigned to the random effects to permit partial pooling, enabling sparsely sampled countries or species to borrow statistical strength from data-rich settings while preserving genuine epidemiological heterogeneity. Because diagnostic misclassification had already been addressed during the Rogan–Gladen adjustment stage, the hierarchical model operated directly on the adjusted prevalence distributions and did not re-apply misclassification modelling in the likelihood, thereby ensuring methodological coherence while maintaining fidelity to established Bayesian misclassification principles [11, 12]. Posterior inference was performed using the No-U-Turn Sampler implemented in PyMC, generating full posterior distributions for global, country-level, and study-level prevalence estimates, as well as posterior predictive distributions for the observed data. Model adequacy was validated using posterior predictive checks comparing simulated and empirical prevalence distributions, together with study-level calibration diagnostics. This hierarchical framework yielded misclassification-adjusted, uncertainty-aware estimates that robustly characterized BEFV prevalence across a diverse evidence base.

### Spatiotemporal modelling of BEFV dynamics

Temporal changes in BEFV exposure were analyzed using a spatiotemporal Bayesian regression model applied to countries with at least two sampling years. Annual prevalence was treated as a binomial outcome, and a non-centred hierarchical formulation was used to stabilize estimation of country-specific temporal slopes. In this structure, each country’s trend was modeled as a deviation from a shared global intercept and slope. Calendar year was centred and scaled for a decade to improve sampler geometry and reduce posterior curvature. The model produced posterior distributions for country-specific intercepts, slopes, and reconstructed annual prevalence estimates, each accompanied by 95% credible intervals. These trajectories enabled direct comparison between empirical observations and inferred long-term patterns in BEFV exposure within each country.

### Temporal trends in diagnostic method usage

To account for the influence of changing diagnostic technologies on reported BEFV prevalence, all studies were reclassified into broad diagnostic families: ELISA versus virus neutralization for serology, and PCR versus virus isolation for active infection detection. The frequency of each method was tabulated by year and aggregated by decade to characterize historical transitions in diagnostic practice. These data were visualized in stacked time-series plots, illustrating the gradual replacement of virus-neutralization and virus-isolation assays by ELISA and PCR, respectively. This contextual analysis supported interpretation of apparent epidemiological fluctuations, especially during periods when the sensitivity of available diagnostic tools differed substantially from contemporary assays.

### Software and reproducibility

All analyses were conducted in R (version 4.3.2) and Python (version 3.11) to ensure reproducibility. Conventional meta-analyses, including random-effects modelling, heterogeneity estimation, and publication bias assessment, were performed in R using the *metafor* (version 4.4-0) and *meta* (version 6.5-0) packages, with graphics generated in *ggplot2* (version 3.4.4). Data preprocessing and restructuring were carried out with *dplyr* (version 1.1.3) and *tidyr* (version 1.3.0). Bayesian hierarchical and spatiotemporal models were implemented in Python using PyMC (version 5.10.4). The No-U-Turn Sampler was executed with four chains, 2,000 warm-up iterations, and 4,000 posterior draws per chain. Convergence and sampler performance were assessed using effective sample size diagnostics and the Gelman–Rubin statistic via *ArviZ* (version 0.17.0). Leave-one-out cross-validation was conducted with Pareto-smoothed importance sampling using *ArviZ*’s integrated functions. All analyses were performed in a controlled computational environment with fixed package versions. Data processing and modelling scripts were organized into reproducible workflows, and the complete codebase will be made available in a version-controlled repository upon publication to facilitate independent verification.

## Results

### Study selection and characteristics

**Fig. 2:**
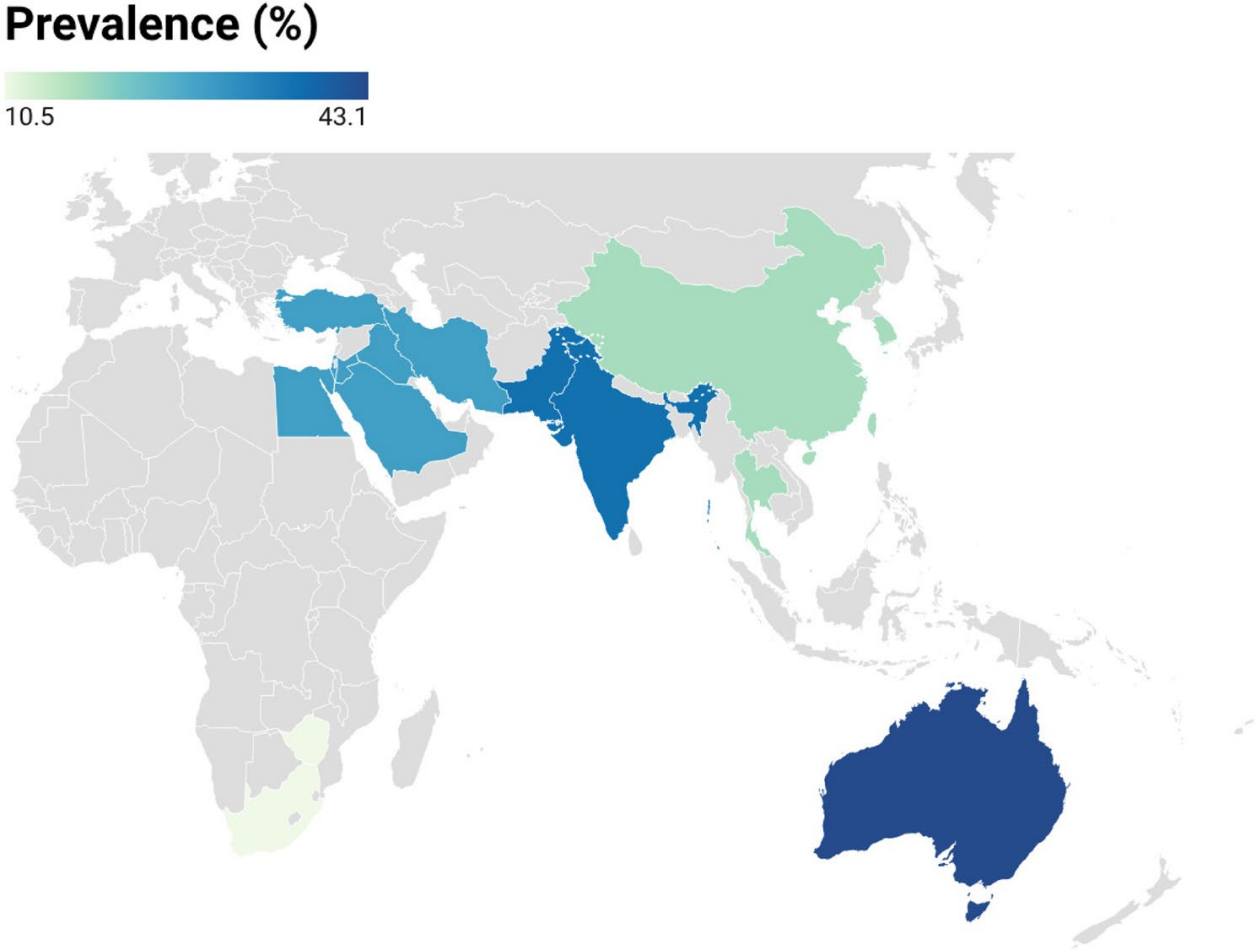
Geographic distribution of observed laboratory-confirmed bovine ephemeral fever virus (BEFV) prevalence in bovine populations (1967–2025). The map displays data derived from 52 studies across 19 countries included in the systematic review, with color intensity indicating reported prevalence (%) ranging from 10.5% to 43.1%. Countries shown in grey had no eligible data available

A total of 52 studies conducted from 1967 to 2025 met the inclusion criteria, representing 19 countries in the systematic review and 16 countries in quantitative modelling, and encompassing 335,754 bovines, of which 69,256 tested positive for BEFV, resulting in a crude prevalence of 20.6%. Laboratory-confirmed studies contributed one or more assay-specific prevalence strata to quantitative meta-analysis and Bayesian modelling. The characteristics of the studies are summarized in Table 1, and regional distributions are presented in Table 2, which summarizes independent extractable prevalence datasets contributing to regional estimates. The Middle East contributed the largest number of datasets (*n* = 24), while East Asia accounted for the greatest sample size (*n* = 9; 241,282 animals). Additional datasets originated from South Asia (*n* = 4), Australia (*n* = 5), and Southern Africa (*n* = 2). Reported prevalence values ranged from 0% to 85.7%, reflecting wide variation in sampling context and diagnostic method. Fig. 2 illustrates the geographical distribution of study locations. The geographic distribution of investigations was uneven, with a concentration in the Middle East and East Asia and comparatively limited representation from South America and parts of Africa.

**Table 2:** Regional summary of BEFV prevalence studies

Region	Countries (Studies)	Total Bovines Tested	Total Positive	Prevalence Range
Middle East	Israel (6), Turkey (8), Saudi Arabia (5), Iran (2), Jordan (1), Iraq (1), Egypt (1)	87,842	25,139	0% – 85.7%
East Asia	China (5), Taiwan (1), South Korea (2), Thailand (1)	241,282	41,885	2.8% – 67.7%
South Asia	Pakistan (3), India (1)	2,586	945	5.2% – 66.0%
Southern Africa	Zimbabwe (1), South Africa (1)	1,403	148	6.0% – 78.4%
Australia	Australia (5)	2,641	1,139	15.6% – 54.5%

Country-level counts represent laboratory-confirmed publications contributing one or more extractable prevalence strata to regional summaries. Publications relying exclusively on clinical diagnosis were included in Table 1 for systematic-review completeness but did not contribute to quantitative regional estimates. Therefore, regional counts do not necessarily correspond to the total number of publications listed in Table 1

### Conventional random-effects meta-analysis

#### Overall prevalence and heterogeneity

The random-effects meta-analysis was conducted on assay-specific prevalence strata derived from the 52 included publications and showed extensive variability in reported BEFV prevalence, with individual study estimates spanning 0% to >80%. The corresponding forest plots appear in Supplementary Figures 1–4 for diagnostic-method subgroups and Supplementary Figures 5–9 for host-species subgroups. Cochran’s Q indicated significant heterogeneity, and both I² and τ² values were high. Across diagnostic subgroups, heterogeneity consistently exceeded I² > 95%, with τ² ranging from 0.025 to 0.150, reflecting marked dispersion in effect sizes across studies.

#### Publication bias (Global Analysis)

**Fig. 3:**
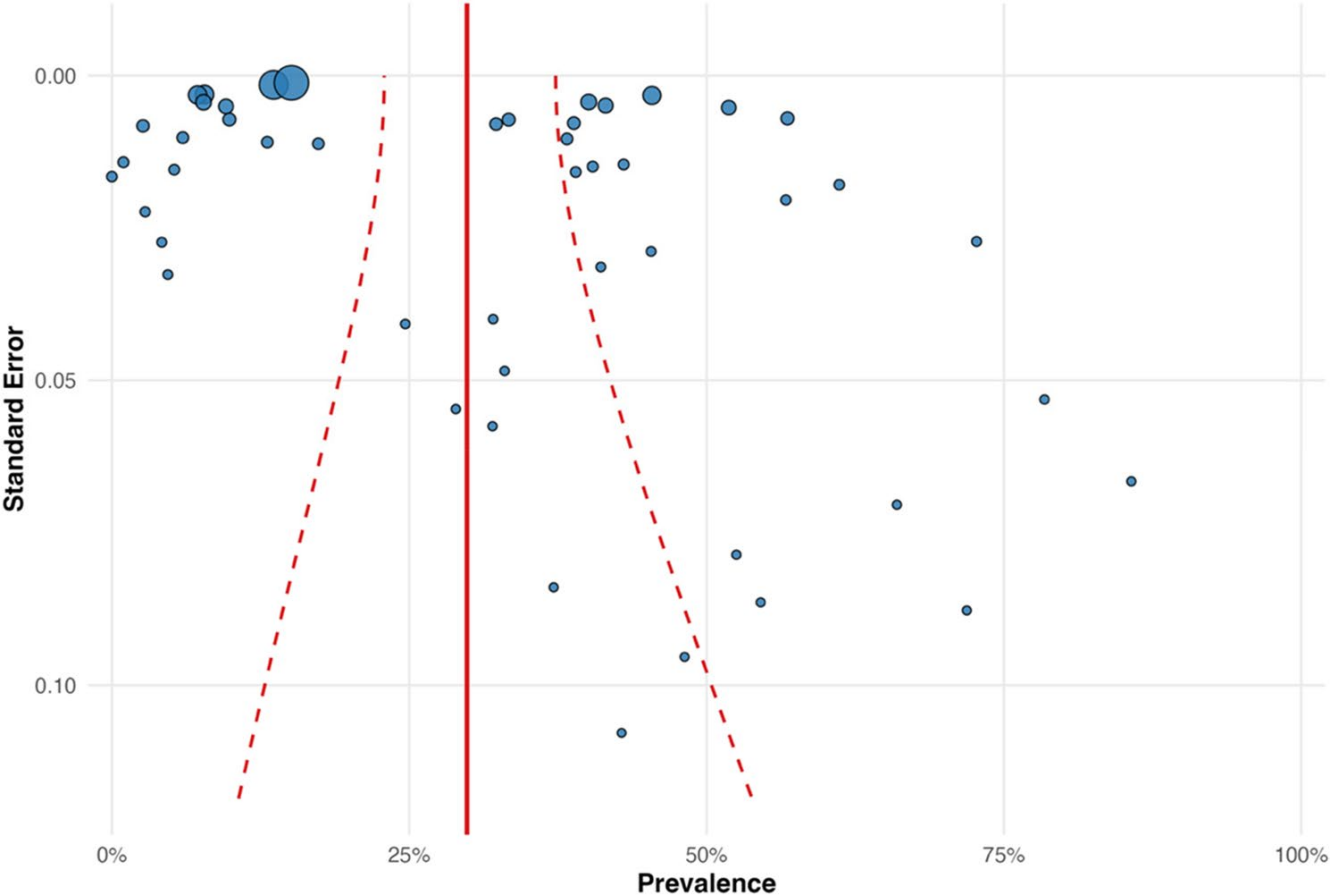
Funnel plot of assay-specific prevalence strata derived from 52 publications

Visual assessment of the funnel plot (Fig. 3) showed mild asymmetry, with fewer small studies reporting low prevalence. Egger’s regression test did not detect statistically significant small-study effects (t = 1.82, df = 46, *p* = 0.075). Given the non-significant result, no trim-and-fill correction was applied at the global level.

### Stratified meta-analysis by diagnostic method

Pooled prevalence estimates differed substantially by diagnostic approach (Table 3). PCR-based studies produced a pooled prevalence of 83.9% (95% CI: 70.5–97.3%) with high heterogeneity (τ² = 0.089; I² = 99.7%). Virus isolation yielded a similar pooled prevalence of 84.2% (59.7–100%), also with extreme heterogeneity (τ² = 0.150; I² = 99.7%). ELISA studies showed a pooled seroprevalence of 65.4% (55.2–75.5%), with lower between-study variance relative to other assays (τ² = 0.025; I² = 94.8%). Virus neutralization tests (VNT) produced the lowest pooled estimate, 37.8% (28.5–47.0%), with substantial heterogeneity (τ² = 0.072; I² = 99.6%). Corresponding forest plots for each diagnostic method are shown in Supplementary Figs. 1–4.

**Table 3:** Subgroup analysis of BEFV prevalence in bovine populations by diagnostic method

Diagnostic Method	k	Total *N*	Events	Pooled Prevalence (%)	95% CI (%)	τ²	I² (%)	*p*-heterogeneity
PCR	20	41,148	8,416	83.9	70.5–97.3	0.089	99.7	< 0.001
ELISA	11	5,504	1,985	65.4	55.2–75.5	0.025	94.8	< 0.001
Virus Neutralization Test (VNT)	33	35,572	11,278	37.8	28.5–47.0	0.072	99.6	< 0.001
Virus Isolation	10	6,008	2,681	84.2	59.7–100	0.150	99.7	< 0.001

*k* number of assay-specific prevalence strata contributing to each subgroup meta-analysis, *Total N* Total cattle tested, *Events* positive cases, *CI* Confidence interval, τ² between-study variance, I² proportion of total variation attributable to heterogeneity

#### Publication bias by diagnostic method

Publication-bias assessment (Table 4) indicated imputed missing studies for ELISA (k = 3), raising the pooled estimate to 71.8% (61.0–82.7%), and for VNT (k = 4), raising the estimate to 42.5% (33.1–51.9%). Egger’s test showed significant asymmetry for VNT (*p* = 0.002) and non-significant results for other assays. PCR and virus isolation groups had no imputed studies.

**Table 4:** Assessment of publication bias by diagnostic method using Egger’s regression, Begg’s rank correlation, and trim-and-fill imputation

Diagnostic Method	Egger’s *p*	Begg’s *p*	k_imputed	Adjusted Prevalence (%)	95% CI (%)
PCR	0.064	0.143	0	—	—
ELISA	0.528	0.718	3	71.8	61.0–82.7
Virus Neutralization Test (VNT)	0.002	0.053	4	42.5	33.1–51.9
Virus Isolation	0.744	0.276	0	—	—

k_imputed = number of imputed missing studies; adjusted prevalence and CI are shown only for subgroups with imputed studies; “—” indicates no imputation or unavailable test result

### Stratified meta-analysis by host species

Subgroup analysis by host species, based on assay-specific strata (Table 5), showed marked variation in pooled BEFV prevalence estimates. Bos taurus exhibited the highest pooled seroprevalence at 77.5% (95% CI: 66.5–88.5%), with very high heterogeneity (I² = 99.3%). *Bos grunniens* (yak) showed a pooled prevalence of 62.4% (95% CI: 49.1–75.7%), based on two studies with substantial heterogeneity (I² = 94.0%). Bubalus bubalis (water buffalo) had a pooled prevalence of 51.3% (95% CI: 43.4–59.3%), with lower but still considerable heterogeneity (I² = 76.0%). Small ruminants showed much lower pooled estimates. Capra hircus (goats) had a prevalence of 21.1% (95% CI: 0.7–41.5%) with high heterogeneity (I² = 86.7%), and Ovis aries (sheep) showed 15.8% (95% CI: −7.6–39.2%), with very high heterogeneity (I² = 97.3%) owing to sparse data. Corresponding forest plots are shown in Supplementary Figs. 5–9.

**Table 5:** Subgroup analysis of BEFV prevalence by host species

Species	k	Total *N*	Events	Pooled Prevalence (%)	95% CI (%)	τ²	I² (%)	*p*-heterogeneity
Bos taurus	25	58,593	24,002	77.5	66.5–88.5	0.076	99.3	< 0.001
Bos grunniens	2	1,406	532	62.4	49.1–75.7	0.009	94.0	< 0.001
Bubalus bubalis	10	926	205	51.3	43.4–59.3	0.012	76.0	< 0.001
Capra hircus	3	319	37	21.1	0.7–41.5	0.026	86.7	0.001
Ovis aries	3	604	34	15.8	0.0–39.2	0.041	97.3	< 0.001

*k* number of assay-specific prevalence strata, *Total N * Total animals tested, *Events* positive cases, *95% CI* 95% Confidence interval, τ² = between-study variance, I² proportion of total variation attributable to heterogeneity

### Publication bias by species

Evaluation of publication bias (Table 6) indicated no evidence of funnel-plot asymmetry across species groups. Egger’s regression tests yielded *p* ≥ 0.152, and Begg’s tests showed *p* ≥ 0.415 where applicable. The trim-and-fill procedure imputed no additional studies (k = 0) for any species subgroup, and pooled estimates remained unchanged.

**Table 6:** Assessment of publication bias by host species using Egger’s regression, Begg’s rank correlation, and trim-and-fill imputation

Species	Egger’s *p*	Begg’s *p*	k-imputed	Adjusted Prevalence (%)	95% CI (%)
Bubalus bubalis	0.244	0.415	0	—	—
Bos taurus	0.379	0.708	0	—	—
Capra hircus	0.152	—	0	—	—
Ovis aries	0.805	—	0	—	—

k_imputed = number of imputed missing studies, “—” indicates no imputation or unavailable test result

### Meta-regression and risk factor analysis

#### Meta-regression

Univariable and multivariable random-effects meta-regression models (Table 7) did not identify any statistically significant moderators of prevalence. The diagnostic method showed p = 0.16–0.81, the host species showed p = 0.10–0.71, and the publication year also showed no significant trend (p = 0.22–0.76). In the multivariable model, overall fit remained non-significant (QM ≈ 4.29; p = 0.16–0.37).

**Table 7:** Uni- and Multivariate Meta-regression of BEFV Prevalence

Term		Univariable			Multivariable		
	k	Prevalence	95% CI	*p*-value	Prevalence	95% CI	*p*-value
Diagnostic method							
Intercept (reference method)		78.5%	70.1–85.0%	–	68.4%	0.0–100.0%	0.159
PCR		60.1%	47.2–71.7%	0.123	60.7%	45.9–73.8%	0.154
Virus Isolation		55.0%	39.8–69.3%	0.521	53.3%	36.2–69.6%	0.712
Virus Neutralization Test (VNT)		–	–	–	36.5%	23.7–51.5%	0.077
Host species							
Intercept (reference species)		57.9%	34.4–78.3%	0.518	–	–	–
Bos grunniens (yak)		75.9%	44.7–92.5%	0.098	67.5%	35.9–88.5%	0.274
Bos taurus (cattle)		72.8%	50.1–87.8%	0.049	64.1%	42.8–81.0%	0.192
Bubalus bubalis (buffalo)		62.5%	35.4–83.5%	0.368	58.1%	34.5–78.5%	0.507
Capra hircus (goat)		54.3%	23.1–82.5%	0.806	54.7%	26.9–79.8%	0.757
Publication year	61	+ 0.00% per year	–	0.215	49.8% (centered at 2012)	49.4–50.1%	0.174

*k* number of assay-specific prevalence strata included in the meta-regression model, *CI* Confidence interval, *PCR* Polymerase chain reaction, *VNT* virus neutralization test

Statistically significant at *p* < 0.05

Within the multivariable model, studies using the virus neutralization test (VNT) had lower estimated prevalence (36.5%, 95% CI: 23.7–51.5%) with a borderline p-value of 0.077, but this did not meet the threshold for statistical significance.

#### Sex as a risk factor

**Fig. 4:**
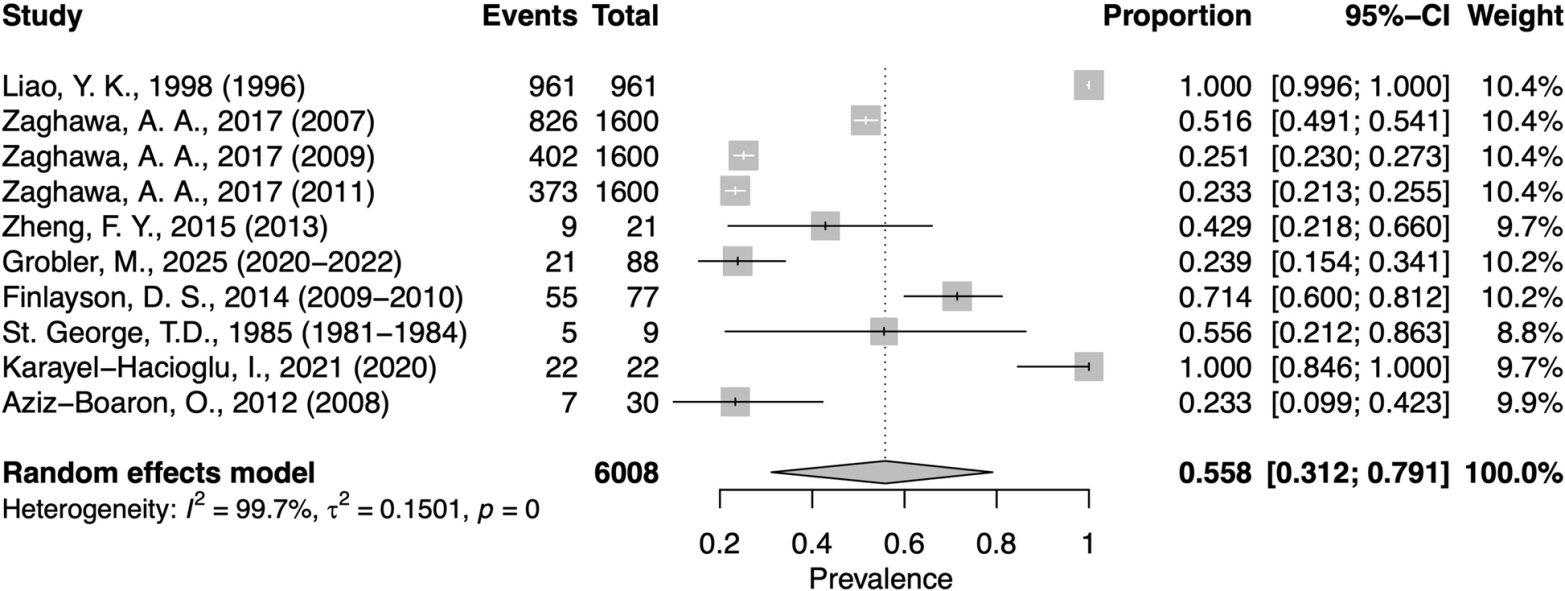
Forest plot of odds ratios for BEFV positivity in males versus females (reference = female)

Sex-specific data from 11 studies yielded a pooled odds ratio of 0.77 (95% CI: 0.40–1.45) for male versus female seropositivity (*p* = 0.412). Heterogeneity was substantial (I² = 88.9%; τ² = 0.9799; *p* < 0.0001). No additional host or management factors could be meta-analyzed due to insufficient reporting. The pooled sex-effects analysis is illustrated in Figure. 4.

### Misclassification-adjusted prevalence (Rogan–Gladen Correction)

#### Adjusted serological prevalence

**Fig. 5:**
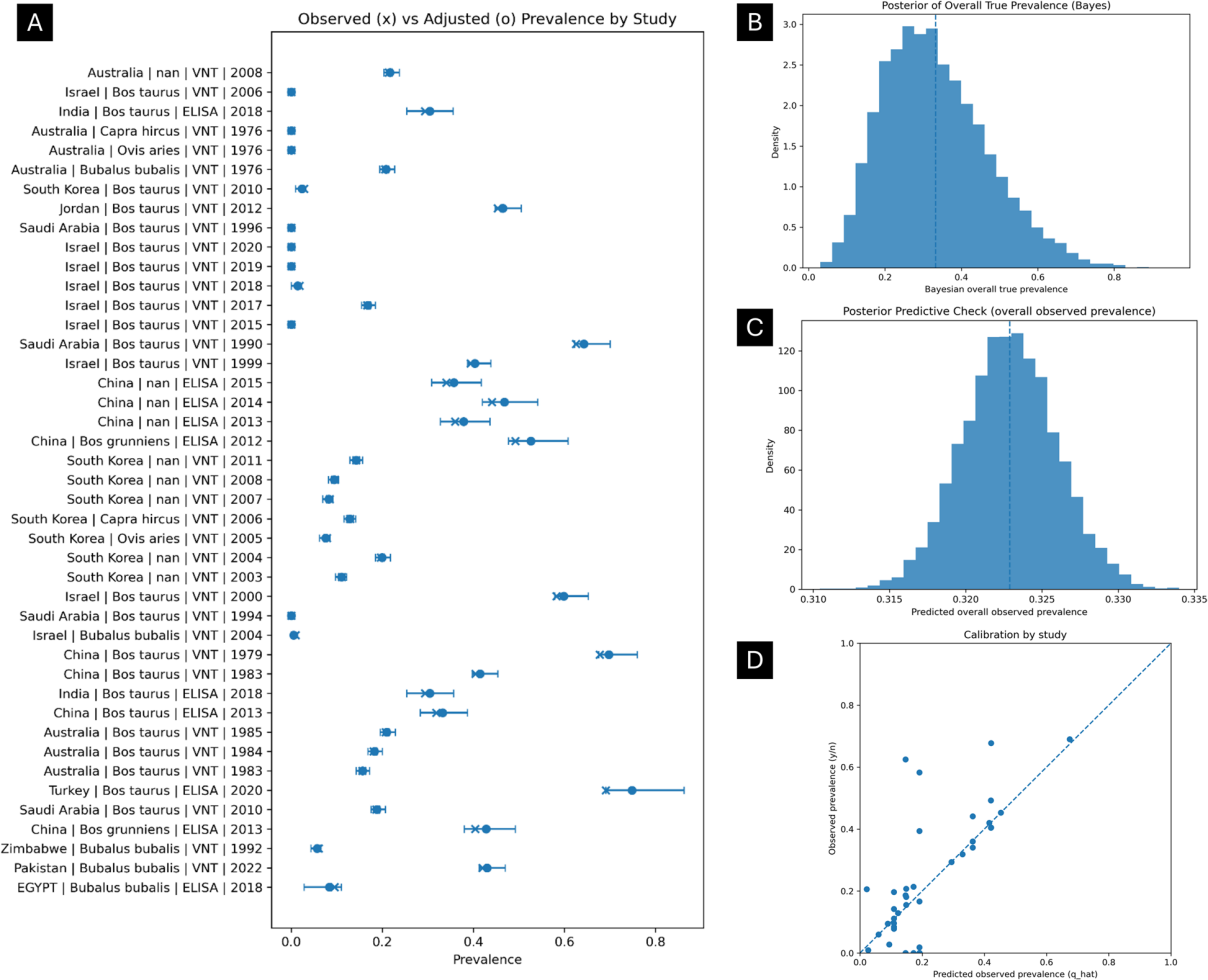
Misclassification-adjusted Bayesian hierarchical model of BEFV seroprevalence. **A** Study-level comparison of observed (×) and misclassification-adjusted (●) prevalence estimates, illustrating the impact of imperfect diagnostic sensitivity and specificity across assays. **B** Posterior distribution of the global true seroprevalence, reflecting uncertainty due to between-study heterogeneity and diagnostic error. **C** Posterior predictive check showing close agreement between simulated and observed overall prevalence. **D** Study-level calibration plot comparing predicted and observed prevalence, demonstrating strong model fit across the full range of estimates

Applying the Rogan–Gladen correction with beta-distributed sensitivity and specificity parameters increased seroprevalence estimates derived from VNT and ELISA studies, as illustrated by the study-level comparisons in Fig. 5A. The global misclassification-adjusted true seroprevalence was 0.332 (95% SI: 0.308–0.376), as shown in the posterior distribution of the hierarchical model (Fig. 5B).

#### Adjusted active-infection prevalencested active-infection prevalence

**Fig. 6:**
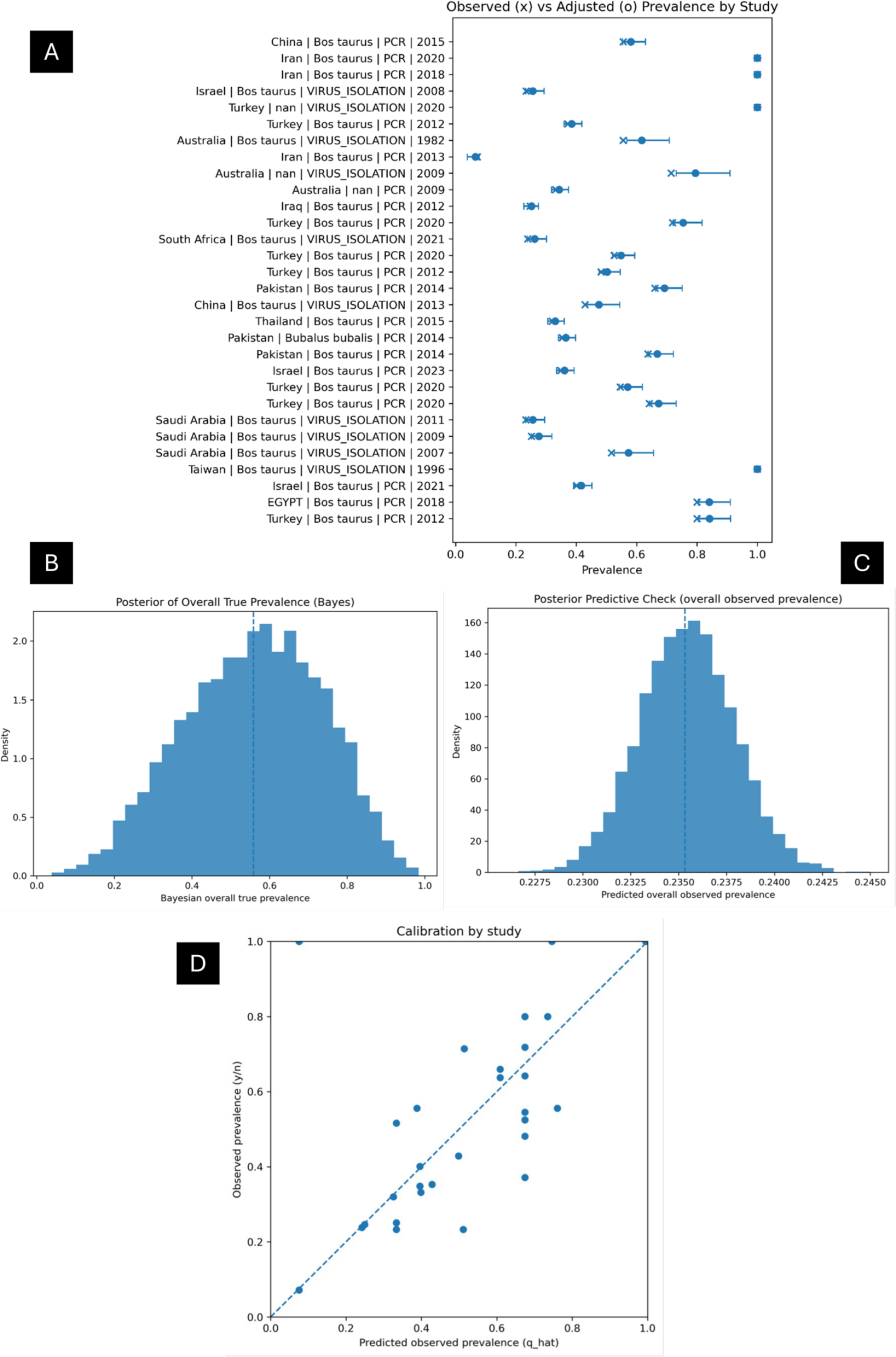
Misclassification-adjusted Bayesian hierarchical model of BEFV active-infection prevalence. **A** Study-level comparison of observed (×) and misclassification-adjusted (●) prevalence estimates for molecular and virus-isolation data, illustrating the impact of imperfect diagnostic sensitivity and specificity. **B** Posterior distribution of the global true active-infection prevalence, reflecting uncertainty due to between-study heterogeneity and diagnostic error. **C** Posterior predictive check demonstrating close agreement between simulated and observed overall prevalence. **D** Study-level calibration plot comparing predicted and observed prevalence, indicating good model fit across the full range of estimates

For molecular and virological detection data, the adjusted global prevalence of active infection was 0.241 (95% SI: 0.226–0.259). Corrections were smaller for molecular data because PCR dominated the dataset and required minimal adjustment, whereas virus isolation contributed fewer studies. The corresponding misclassification-adjusted active-infection distributions are shown in Fig. 6A–B, with country- and study-level adjustments provided in Supplementary Table 2.

### Bayesian hierarchical model: global and country-level prevalence

#### Global active-infection prevalence

The Bayesian hierarchical model applied to PCR and virus-isolation data yielded a higher global posterior mean active-infection prevalence of 0.56 (95% CrI: 0.27–0.87). The wider uncertainty reflects substantial between-study and between-country variability and the narrow temporal window of detectable viremia. Posterior predictive checks demonstrated close agreement between simulated and observed detection proportions (Fig. 6C), and study-level calibration diagnostics are presented in Fig. 6D.

This hierarchical estimate exceeds the misclassification-adjusted value (0.241) because the model incorporates country- and study-level random effects and estimates latent prevalence under partial pooling rather than the raw frequency of positives. During outbreaks, when detection rates are high, the hierarchical framework concentrates PCR sampling within epidemic clusters, which increases the posterior mean relative to the globally averaged misclassification-adjusted value. Accordingly, the 0.56 estimate reflects the outbreak-driven structure of the available PCR evidence.

### Country-level estimates of active infection

Country-specific posterior means, presented in Supplementary Table 2, showed marked geographic differences. Taiwan exhibited the highest estimated prevalence (0.997, CrI 0.987–1.000), followed by Egypt (0.914, CrI 0.647–0.999), Turkey (0.812, CrI 0.467–0.998), and Pakistan (0.670, CrI 0.354–0.905). Lower estimates were observed in Iran (0.060, CrI 0.012–0.170) and South Africa (0.166, CrI 0.036–0.414).

#### Characteristics of active-infection variability

Across countries, posterior distributions showed wide credible intervals and substantial variability, reflecting the short duration of PCR-detectable infection and the concentration of sampling during documented epidemic periods. The resulting posterior distributions describe a landscape of sporadic but intense viral circulation, with high peaks during outbreaks and negligible detection between them.

### Spatiotemporal trends in seroprevalence and active infection

**Fig. 7:**
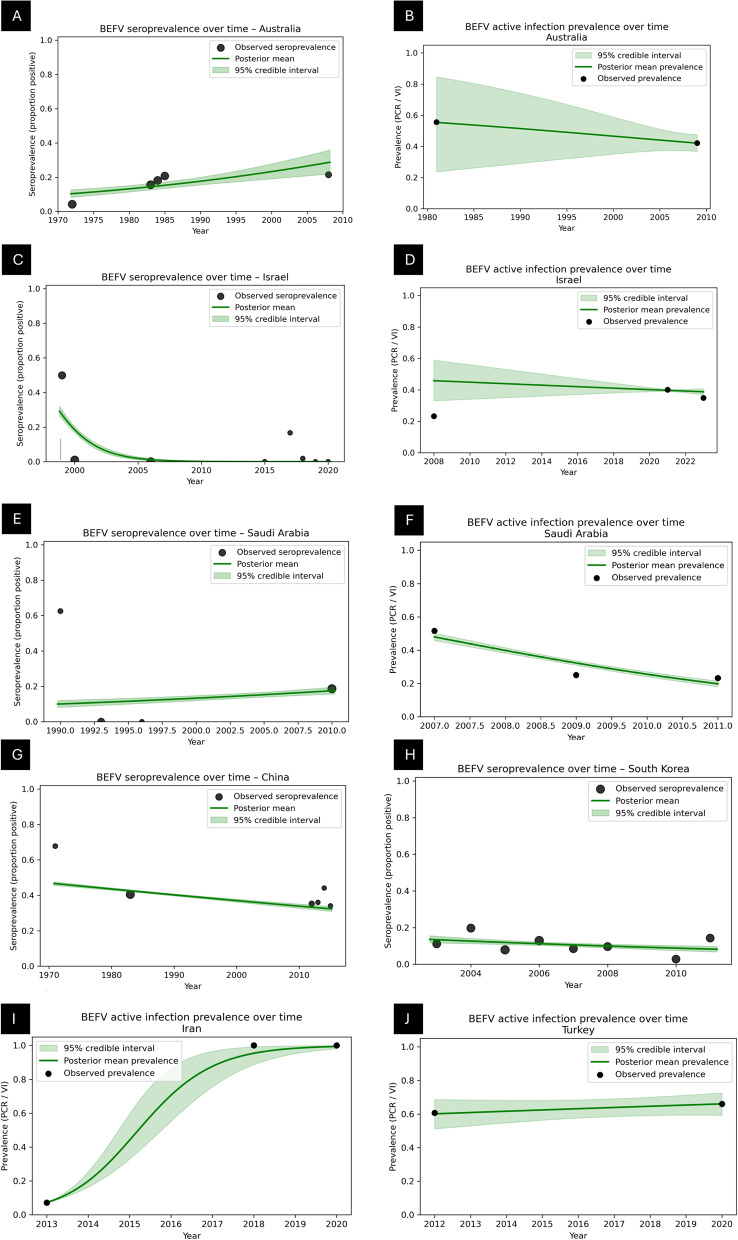
Country-level temporal patterns in BEFV serological exposure and active infection. Posterior estimates from Bayesian spatiotemporal models illustrate heterogeneous epidemiological trajectories across countries. Seroprevalence trends are shown for Australia (**A**), Israel (**C**), Saudi Arabia (**E**), China (**G**), and South Korea (**H**), while active-infection prevalence based on PCR or virus isolation is shown for Australia (**B**), Israel (**D**), Saudi Arabia (**F**), Iran (**I**), and Turkey (**J**). Australia, China, Israel, and South Korea exhibited declining seroprevalence over time, consistent with reduced widespread transmission. Saudi Arabia maintained low baseline immunity with decreasing active infection. Iran showed persistently high and increasing PCR positivity, suggesting sustained endemic circulation, whereas Turkey displayed elevated outbreak-associated active infection prevalence despite limited longitudinal data. Solid lines represent posterior means and shaded areas indicate 95% credible intervals that incorporate diagnostic uncertainty and study-level heterogeneity

The spatiotemporal Bayesian models identified clear temporal variation in BEFV exposure and active infection across countries (Fig. 7A–J). Trends differed by setting and diagnostic dataset but were internally consistent with available longitudinal observations.

#### Australia

Serological evidence from Australia (Fig. 7A) indicates consistently low BEFV exposure across five decades, with most estimates ranging between 10% and 25% and no indication of a sustained long-term increase. Although several outbreak investigations documented short-lived surges in clinical cases or virological detection, these events did not produce lasting rises in population-level immunity. Active-infection prevalence (Fig. 7B) was highest during early outbreak years but declined in more recent surveys, reflecting a pattern of sporadic, episodic incursions rather than stable endemic transmission.

#### Israel

Israel showed a marked temporal decline. Active-infection estimates (Fig. 7D) decreased after 2008, and seroprevalence (Fig. 7C) fell sharply from high values in the late 1990s to near zero throughout much of the 2000s. Minor increases in later years were also detected. The combined outputs indicate prolonged periods of very low circulation.

#### Saudi Arabia

In Saudi Arabia, active-infection prevalence declined between approximately 2007 and 2011 (Fig. 7F). In contrast, seroprevalence increased over time (Fig. 7E), although with wide variability across studies. This divergence reflects differing temporal patterns across diagnostic datasets.

#### China

Long-term serological data from China (Fig. 7G) showed a pronounced downward trajectory. Surveys from the 1970–1980 s reported high seroprevalence, whereas post-2010 studies consistently produced lower values (~ 0.33–0.45). The temporal model captured this sustained decline in cumulative exposure.

#### South Korea

South Korea exhibited low-to-moderate seroprevalence between 2003 and 2011 (Fig. 7H). The temporal model indicated a slight downward trend with narrow uncertainty. In the absence of molecular data, available serology suggests stable, low-level circulation during the sampling period.

#### Iran

Iran showed a distinct rise in active infection (Fig. 7I). PCR data revealed a sharp increase during the mid-2010s. Serological information was limited, but available values aligned with the period of elevated molecular detection, indicating recent increases in exposure.

#### Turkey

Turkey showed an upward trend in active-infection prevalence between 2012 and 2020 (Fig. 7J), though estimates carried substantial uncertainty due to limited sampling. Serological studies indicated relatively high baseline exposure. Together, the datasets reflected recurrent BEFV activity recently.

### Temporal evolution of diagnostic methods across five decades

**Fig. 8:**
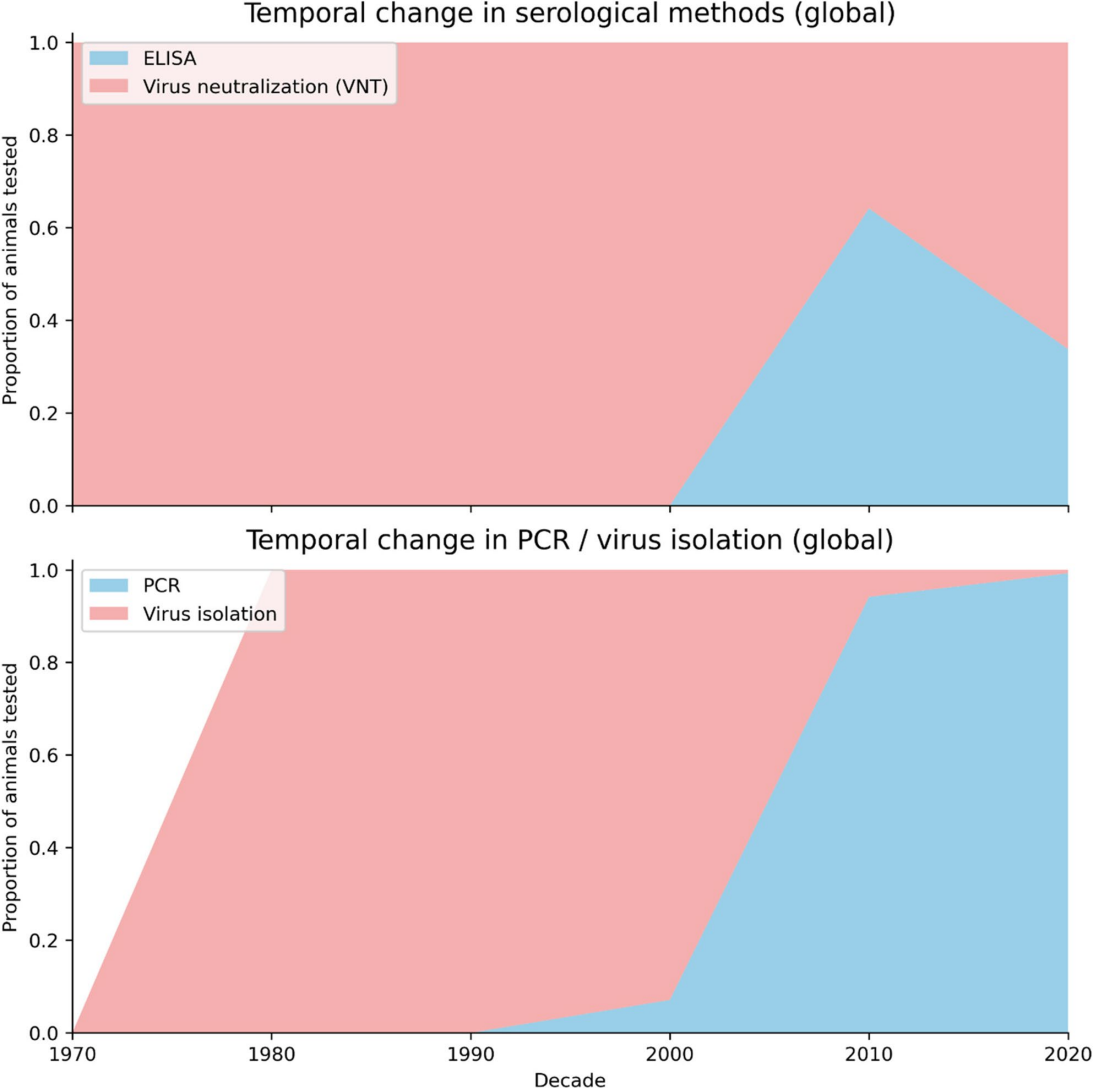
Temporal evolution of diagnostic methods used for BEFV detection by decade. Stacked areas represent the proportion of animals tested within each decade according to diagnostic method. The upper panel shows the distribution of serological assays, comparing virus neutralization tests and enzyme-linked immunosorbent assays. The lower panel shows methods used for detection of active infection, comparing virus isolation and polymerase chain reaction. Data are aggregated by decade based on the reported sampling period of each study. The figure illustrates the progressive transition from virus neutralization and virus isolation in earlier decades toward ELISA and PCR in more recent decade

The distribution of diagnostic methods used in BEFV surveillance changed markedly from 1967 to 2025 (Fig. 8). Early studies relied almost exclusively on virus neutralization tests (VNT) for serology and virus isolation for detecting active infection. These methods dominated surveillance through the 1970s and 1980s and represent the primary diagnostic basis of early prevalence estimates.

Since the 1990s, ELISA assays have grown in popularity. Their launch coincided with a gradual decrease in the usage of VNT and a significant increase in ELISA-based serological testing. By the 2000s, ELISA had effectively supplanted VNT for routine surveillance.

A second major transition occurred with the expansion of molecular diagnostics after the early 2000s. PCR, initially limited to research contexts, increased rapidly and became the predominant method for detecting active infections by the 2010s. Virus isolation declined sharply during this period and became largely restricted to confirmatory or specialist virological investigations.

By the end of the study period, BEFV diagnostics had converged toward ELISA for antibody detection and PCR for active infection. This evolution in methodology resulted in substantial differences in diagnostic sensitivity across decades, illustrating the importance of a misclassification adjustment when comparing historical prevalence estimates.

## Discussion

This study provides the first global, misclassification-adjusted estimate of bovine ephemeral fever (BEF) seroprevalence and infection prevalence, offering a quantitative foundation for understanding the true epidemiological burden of the disease. By synthesizing data from 52 studies across 19 countries in the systematic review, with 16 countries contributing to quantitative modelling, and applying a Bayesian hierarchical model that explicitly corrects for imperfect diagnostic sensitivity and specificity, we estimated a global true seroprevalence of 0.33 and a misclassification-adjusted active infection prevalence of 0.241. These results reveal striking geographical heterogeneity: countries such as Pakistan and Turkey exhibit posterior seroprevalence values nearing 0.84, whereas regions like Israel and Australia maintain substantially lower baseline immunity. In the context of recent literature framing BEF as an emerging global threat [1], our findings suggest that reliance on unadjusted serological and molecular data may have underestimated transmission intensity. By correcting diagnostic misclassification and reconstructing country-specific temporal trends, this work represents the largest BEF meta-analysis to date and provides a robust statistical foundation for guiding surveillance priorities, outbreak preparedness, and targeted control strategies.

Previous reviews have characterized BEF as a climate-sensitive, transboundary, vector-borne disease capable of producing 80–100% herd-level morbidity, yet global assessments have remained largely qualitative and lacked standardized or quantitative measures of exposure. While Pyasi et al. emphasize BEF’s severity, they do not provide epidemiological metrics that quantify true burden [1]. Our analysis directly addresses this gap by applying a misclassification-adjusted Bayesian framework to 335,754 animals across 16 countries, yielding the first calibrated global estimates of BEF exposure and infection. These values refine earlier impressions by confirming exceptionally high burdens in Pakistan and Turkey while simultaneously revealing markedly lower immunity in Israel and Australia. By shifting BEF research from descriptive narrative to actionable epidemiological evidence, our results establish a reliable quantitative baseline for international disease preparedness and risk stratification.

Regional differences in BEF epidemiology reflect the intricate interplay of climate, vector ecology, host immunity, and viral evolution. High-prevalence countries such as Pakistan and Turkey, with seroprevalence values approaching 0.84, reflect subtropical environments that support prolonged *Culicoides* vector activity and dense livestock populations that sustain endemic transmission. Seasonal rainfall patterns, temperature regimes, and irrigation practices in these regions likely enhance vector breeding habitats and extend transmission windows. Intermediate regions, such as Egypt and China, display fluctuating or declining seroprevalence (≈ 0.33–0.45) even as genomic diversification continues; Chen et al. documented a novel BEFV sub-lineage and recombination in China, demonstrating that evolutionary potential persists despite waning historical exposure [58]. Conversely, low-prevalence regions such as Israel (0.15) and Australia (0.12) remain highly outbreak-prone, as illustrated by recurrent Israeli epizootics culminating in 2023, during which BEF incidence exceeded bluetongue virus by a factor of 2.21 [29]. These findings underscore how shifting combinations of immunity, ecological variability, and viral evolution drive the observed heterogeneity in BEF transmission across regions.

Long-standing diagnostic heterogeneity has further complicated BEF epidemiology. Serological assays display wide variability in sensitivity and specificity, and early PCR methods often targeted variable genomic regions vulnerable to lineage-associated detection failure. Golender et al. demonstrated that modern N-gene real-time RT-qPCR assays achieve ~ 98% sensitivity and detect positives missed by older G-gene assays, confirming that earlier surveillance likely underestimated true infection rates [59]. By incorporating misclassification adjustment into our Bayesian model, we reconciled inflated outbreak-associated PCR prevalence (83.9%) with a more plausible global active-infection estimate of 0.241. As diagnostic technologies continue to advance, particularly in regions with substantial genetic diversity, our approach ensures that prevalence estimates remain coherent, robust, and reflective of true viral transmission rather than methodological artifacts. Continued improvements in assay sensitivity, genomic target selection, and international standardization are expected to further refine BEF prevalence estimates and reduce residual uncertainty in future epidemiological assessments.

The epidemiological patterns quantified here align with substantial economic consequences documented in affected regions. Lavon et al. reported herd-level morbidity ranging from 10% to 90.7% during Israel’s 2021 outbreak, with affected cows losing 14–16 kg of milk per day and farms incurring more than USD 100,000 in direct losses during a single outbreak cycle [3]. Such impacts highlight the vulnerability of susceptible populations and illustrate that outbreaks in high-prevalence regions like Pakistan and Turkey where the force of infection is greater and cattle densities are higher are likely to generate even more severe economic disruption. By linking epidemiological burden with real-world production losses, this analysis reinforces the need for proactive, risk-based interventions to mitigate both health and financial impacts.

Mechanistic insights from genomic and diagnostic research help explain the spatial and temporal heterogeneity captured in our model. BEFV continues to evolve across regions, with Chen et al. identifying novel sub-lineages and recombination events in China [58], while Golender et al. showed that Israel’s 2023 outbreak was driven by a “*Mayotte-like*” strain distinct from earlier local viruses-evidence of lineage turnover and cross-regional introductions [29]. These evolutionary processes influence susceptibility profiles and outbreak severity, particularly in regions with low baseline immunity. Advances in diagnostic sensitivity also shape surveillance outcomes: older G-gene PCRs may have missed variant strains, whereas newer N-gene assays detect a broader lineage spectrum [59]. Together, these mechanistic layers viral evolution, vector ecology, host immunity, and diagnostic performance explain the heterogeneity observed across countries and reinforce the need for integrated genomic–epidemiological approaches.

These findings carry important implications for BEF control and future research. Harmonized, high-sensitivity molecular diagnostics capable of detecting diverse BEFV lineages are essential to improving surveillance quality and minimizing misclassification. Routine genomic monitoring is needed to track lineage diversification, recombination, and transboundary introductions, which have demonstrable effects on outbreak dynamics. Country-level seroprevalence estimates can guide risk-based vaccination and vector-control programs, prioritize high-prevalence regions such as Pakistan and Turkey while strengthening early-warning systems in low-immunity but outbreak-prone settings like Israel and Australia. Given the substantial economic losses associated with BEF, proactive interventions are likely to be cost-effective. Continued progress will require integrating genomic and epidemiological datasets, expanding climate-driven modeling to account for shifting *Culicoides* ecologies, and standardizing diagnostic and sampling methodologies to enhance comparability and reduce heterogeneity across studies.

Despite these strengths, this study has several limitations. Geographic gaps, particularly in South America and parts of Africa, restrict comprehensive global inference. Future epidemiological and molecular surveillance studies in these underrepresented regions are needed to clarify transmission dynamics and improve the representativeness of global burden estimates. Incomplete reporting of diagnostic test characteristics in primary studies introduces residual uncertainty despite misclassification adjustment. The concentration of studies in specific regions such as the Middle East and East Asia may also introduce regional sampling bias into pooled estimates. In addition, several epidemiological investigations identified through the systematic search relied exclusively on clinical diagnosis of BEF without laboratory confirmation; although these studies were retained for descriptive completeness in the systematic review, they were excluded from quantitative prevalence estimation and Bayesian modelling (Supplementary Table 4), which may have reduced geographic or temporal coverage of the meta-analysis. Temporal coverage was uneven across countries, limiting the precision of long-term trend reconstruction, and heterogeneity in sampling frames and study designs remains unavoidable in meta-analyses. Nevertheless, the Bayesian hierarchical framework employed here, incorporating explicit modelling of diagnostic error and random-effects structures, mitigates these constraints by appropriately propagating uncertainty and producing robust, generalizable estimates that substantially advance understanding of BEF epidemiology.

## Conclusion

This study provides the first global, misclassification-adjusted estimates of BEF seroprevalence and active infection prevalence, revealing substantially greater and more geographically heterogeneous transmission than previously recognized. By integrating data from 52 studies across 19 countries in the systematic review, with 16 countries included in quantitative modelling within a Bayesian hierarchical framework, we demonstrate exceptionally high burdens in Pakistan and Turkey and persistent outbreak vulnerability in low-immunity settings such as Israel and Australia. These findings highlight the urgent need for harmonized, high-sensitivity diagnostic platforms and standardized surveillance protocols to ensure accurate detection across diverse viral lineages and ecological contexts. Targeted vaccination strategies and strengthened vector-control programs should be prioritized in high-prevalence and outbreak-prone regions, while low-immunity countries require enhanced early-warning systems to mitigate epidemic resurgence. Importantly, the calibrated country-level estimates generated here provide a quantitative foundation to guide evidence-based policy decisions, optimize surveillance investment, and allocate limited veterinary public health resources according to regional risk profiles. Despite remaining geographic and methodological gaps, this work establishes a robust global baseline for future epidemiological, genomic, and climate-informed modelling efforts aimed at reducing the expanding impact of BEF.

## Supplementary Information

Supplementary Material 1.

## Data Availability

The datasets supporting the conclusions of this article are included within the article and its supplementary files. The analytical code used for the Bayesian analyses is available from the corresponding author upon reasonable request.
