# Supplementary material for: Global prevalence and epidemiological dynamics of bovine ephemeral fever: a misclassification-adjusted bayesian meta-analysis
Source: BMC Vet Res. 2026 May 4;22:364. doi: 10.1186/s12917-026-05529-1 (PMC13289255; doi:10.1186/s12917-026-05529-1)
Supplement: Supplementary file 1 — Supplementary Material 1. [file 12917_2026_5529_MOESM1_ESM.docx]

**Appendix 1
----------------------------------------------------------------------------------------------------------------------------------**

**PubMed Search Strategy**

| **Line** | **Search terms** | **Notes** |
| --- | --- | --- |
| 1 | "Ephemeral Fever, Bovine"[Mesh] OR "Ephemeral Fever"[Mesh] | MeSH terms for BEFV |
| 2 | "Bovine ephemeral fever"[tiab] OR BEFV[tiab] OR "three-day sickness"[tiab] OR "ephemeral fever virus"[tiab] OR "bovine ephemeral fever virus"[tiab] OR "ephemeral fever"[tiab] OR "BEF"[tiab] OR "ephemeral fever outbreak"[tiab] | Free-text terms/synonyms for BEFV |
| 3 | #1 OR #2 | Combine MeSH and free-text for disease |
| 4 | "Prevalence"[Mesh] OR "Epidemiology"[Mesh] OR "Incidence"[Mesh] OR "Outbreaks"[Mesh] OR "Seroprevalence"[Mesh] | MeSH terms for prevalence/epidemiology |
| 5 | prevalence[tiab] OR seroprevalence[tiab] OR incidence[tiab] OR epidemiology[tiab] OR outbreak*[tiab] OR surveillance[tiab] OR seroepidemiology[tiab] OR "sero-prevalence"[tiab] OR "sero epidemiology"[tiab] OR burden[tiab] OR distribution[tiab] | Free-text terms for prevalence/epidemiology |
| 6 | #4 OR #5 | Combine MeSH and free-text for outcome |
| 7 | #3 AND #6 | Final combined search: disease AND prevalence/epidemiology |
| 8 | "Animals"[Mesh] | Optional filter: animal studies only |
| 9 | #7 AND #8 | Final animal-only search (if needed) |

**Ovid MEDLINE Search Strategy**

| **Line** | **Search terms** | **Notes** |
| --- | --- | --- |
| 1 | exp Ephemeral Fever, Bovine/ | MeSH explosion for BEFV |
| 2 | "Bovine ephemeral fever".ti,ab. OR BEFV.ti,ab. OR "three-day sickness".ti,ab. OR "ephemeral fever virus".ti,ab. OR "bovine ephemeral fever virus".ti,ab. OR "ephemeral fever".ti,ab. OR BEF.ti,ab. OR "ephemeral fever outbreak".ti,ab. | Free-text terms/synonyms for BEFV in title/abstract |
| 3 | 1 OR 2 | Combine MeSH and free-text for disease |
| 4 | exp Prevalence/ OR exp Epidemiology/ OR exp Incidence/ OR exp Outbreaks/ OR exp Seroprevalence/ | MeSH terms for prevalence/epidemiology |
| 5 | prevalence.ti,ab. OR seroprevalence.ti,ab. OR incidence.ti,ab. OR epidemiology.ti,ab. OR outbreak*.ti,ab. OR surveillance.ti,ab. OR seroepidemiology.ti,ab. OR "sero-prevalence".ti,ab. OR "sero epidemiology".ti,ab. OR burden.ti,ab. OR distribution.ti,ab. | Free-text for prevalence/epidemiology |
| 6 | 4 OR 5 | Combine MeSH and free-text for outcome |
| 7 | 3 AND 6 | Final search: BEFV AND prevalence/epidemiology |
| 8 | Animals/ | Optional: restrict to animal studies |
| 9 | 7 AND 8 | Final animal-only search (if needed) |

- exp = explode MeSH term (includes all narrower terms)
- .ti,ab. = searches title and abstract
- * = truncation to include multiple word endings (e.g., outbreak/outbreaks)

**Ovid EMBASE Search Strategy**

| **Line** | **Search terms** | **Notes** |
| --- | --- | --- |
| 1 | 'bovine ephemeral fever'/exp | Emtree term (exploded) for BEFV |
| 2 | 'bovine ephemeral fever':ti,ab OR BEFV:ti,ab OR 'three-day sickness':ti,ab OR 'ephemeral fever virus':ti,ab OR 'bovine ephemeral fever virus':ti,ab OR 'ephemeral fever':ti,ab OR BEF:ti,ab OR 'ephemeral fever outbreak':ti,ab | Free-text terms/synonyms in title/abstract |
| 3 | 1 OR 2 | Combine Emtree and free-text for disease |
| 4 | 'prevalence'/exp OR 'epidemiology'/exp OR 'incidence'/exp OR 'outbreak'/exp OR 'seroprevalence'/exp | Emtree terms for prevalence/epidemiology |
| 5 | prevalence:ti,ab OR seroprevalence:ti,ab OR incidence:ti,ab OR epidemiology:ti,ab OR outbreak*:ti,ab OR surveillance:ti,ab OR seroepidemiology:ti,ab OR 'sero-prevalence':ti,ab OR 'sero epidemiology':ti,ab OR burden:ti,ab OR distribution:ti,ab | Free-text terms for prevalence/epidemiology |
| 6 | 4 OR 5 | Combine Emtree and free-text for outcome |
| 7 | 3 AND 6 | Final combined search: BEFV AND prevalence/epidemiology |
| 8 | 'animal'/exp | Optional: limit to animal studies |
| 9 | 7 AND 8 | Final animal-only search (if needed) |

- /exp = explode Emtree term (includes all narrower terms)
- :ti,ab = search title and abstract
- * = truncation for multiple endings (e.g., outbreak/outbreaks)
- Single quotes ' ' required around multi-word terms in Ovid EMBASE

**Scopus Search Strategy**

| **Line** | **Search terms** | **Notes** |
| --- | --- | --- |
| 1 | TITLE-ABS-KEY("Bovine ephemeral fever") OR TITLE-ABS-KEY(BEFV) OR TITLE-ABS-KEY("three-day sickness") OR TITLE-ABS-KEY("ephemeral fever virus") OR TITLE-ABS-KEY("bovine ephemeral fever virus") OR TITLE-ABS-KEY("ephemeral fever") OR TITLE-ABS-KEY(BEF) OR TITLE-ABS-KEY("ephemeral fever outbreak")) | Disease/virus terms (broader synonyms for BEFV) |
| 2 | TITLE-ABS-KEY(prevalence) OR TITLE-ABS-KEY(seroprevalence) OR TITLE-ABS-KEY(incidence) OR TITLE-ABS-KEY(epidemiology) OR TITLE-ABS-KEY(outbreak*) OR TITLE-ABS-KEY(surveillance) OR TITLE-ABS-KEY(seroepidemiology) OR TITLE-ABS-KEY("sero-prevalence") OR TITLE-ABS-KEY("sero epidemiology") OR TITLE-ABS-KEY(burden) OR TITLE-ABS-KEY(distribution) | Prevalence/epidemiology terms |
| 3 | 1 AND 2 | Combine disease AND prevalence/epidemiology |
| 4 | LIMIT 3 TO (ANIMALS) | Optional: limit to animal studies |
| 5 | LIMIT 3 TO (ALL) | If no species restriction, use this |

• * in outbreak* captures outbreak/outbreaks

**Web of Science Search Strategy**

| **Line** | **Search terms** | **Notes** |
| --- | --- | --- |
| 1 | TS=("Bovine ephemeral fever" OR BEFV OR "three-day sickness" OR "ephemeral fever virus" OR "bovine ephemeral fever virus" OR "ephemeral fever" OR BEF OR "ephemeral fever outbreak") | Disease/virus terms (broader synonyms for BEFV) |
| 2 | TS=(prevalence OR seroprevalence OR incidence OR epidemiology OR outbreak* OR surveillance OR seroepidemiology OR "sero-prevalence" OR "sero epidemiology" OR burden OR distribution) | Prevalence/epidemiology terms |
| 3 | #1 AND #2 | Combine disease AND prevalence/epidemiology |
| 4 | Refine by: Document Type = Article OR Review | Optional: limit to peer-reviewed research and reviews |
| 5 | Refine by: Species = Animals | Optional: animal studies only |

- **TS=** searches title, abstract, author keywords, and Keywords Plus®.
- * in outbreak* captures both “outbreak” and “outbreaks.”
- No language or publication date limits applied initially, for maximal coverage.

**CAB Abstracts (CABI) Search Strateg**

| **Line** | **Search terms** | **Notes** |
| --- | --- | --- |
| 1 | MAINSUBJECT("Bovine ephemeral fever") | CAB Thesaurus term for BEFV |
| 2 | TI,AB("Bovine ephemeral fever" OR BEFV OR "three-day sickness" OR "ephemeral fever virus" OR "bovine ephemeral fever virus" OR "ephemeral fever" OR BEF OR "ephemeral fever outbreak") | Free-text terms in title/abstract |
| 3 | 1 OR 2 | Combine controlled vocabulary and free-text for disease |
| 4 | MAINSUBJECT("Prevalence") OR MAINSUBJECT("Epidemiology") OR MAINSUBJECT("Incidence") OR MAINSUBJECT("Outbreaks") OR MAINSUBJECT("Seroprevalence") | CAB Thesaurus terms for prevalence/epidemiology |
| 5 | TI,AB(prevalence OR seroprevalence OR incidence OR epidemiology OR outbreak* OR surveillance OR seroepidemiology OR "sero-prevalence" OR "sero epidemiology" OR burden OR distribution) | Free-text for prevalence/epidemiology |
| 6 | 4 OR 5 | Combine CAB Thesaurus and free-text for outcome |
| 7 | 3 AND 6 | Final combined search: disease AND prevalence/epidemiology |
| 8 | MAINSUBJECT("Animals") | Optional: limit to animal studies |
| 9 | 7 AND 8 | Final animal-only search (if needed) |

- **MAINSUBJECT** = searches CAB Thesaurus (controlled vocabulary)
- TI,AB = searches title and abstract
- * in outbreak* captures multiple word endings (e.g., outbreak/outbreaks)
- No language or publication date limits applied initially

**Supplementary Table 1. Quality Assessment of BEFV Prevalence Studies Using a Modified NOS Tool**

| **First Author, Year** | **S1- Sampling Frame Representativeness (0/1)** | **S2- Clear Inclusion / Exclusion Criteria (0/1)** | **S3- Adequate Sample Size Reported (0/1)** | **C1- Validated Diagnostic Test Used (0/1)** | **C2- Diagnostic Protocol & Cut-offs Reported (0/1)** | **O1- Clear Numerator / Denominator (0/1)** | **O2- Outcome Reporting Reproducible (0/1)** | **QA Score (0–7)** | **Risk of Bias (Low / Moderate / High)** |
| --- | --- | --- | --- | --- | --- | --- | --- | --- | --- |
| **Tonbak, 2013** | 1 | 0 | 1 | 1 | 0 | 1 | 0 | **4** | **Moderate** |
| **El-Allawy, 2021** | 1 | 1 | 1 | 1 | 1 | 1 | 1 | **7** | **Low** |
| **Nadeem, 2024** | 1 | 1 | 1 | 1 | 1 | 1 | 1 | **7** | **Low** |
| **Anderson, 1998** | 0 | 0 | 0 | 0 | 0 | 1 | 1 | **2** | **High** |
| **Liu, 2016** | 1 | 1 | 1 | 1 | 1 | 1 | 1 | **7** | **Low** |
| **Lavon, 2023** | 1 | 1 | 1 | 1 | 1 | 1 | 1 | **7** | **Low** |
| **Liao, 1998** | 0 | 0 | 0 | 0 | 0 | 1 | 1 | **2** | **High** |
| **Wang, 2001** | 1 | 0 | 1 | 1 | 0 | 1 | 0 | **4** | **Moderate** |
| **Zaghawa, 2017** | 1 | 1 | 1 | 1 | 1 | 1 | 0 | **6** | **Low** |
| **Hsieh, Y. C,2005** | 1 | 0 | 1 | 1 | 0 | 1 | 0 | **4** | **Moderate** |
| **Islam, 2018** | 1 | 1 | 1 | 1 | 1 | 1 | 1 | **7** | **Low** |
| **Zaghawa, 2016** | 1 | 1 | 1 | 1 | 0 | 1 | 1 | **6** | **Low** |
| **Tokgoz, 2023** | 1 | 1 | 1 | 1 | 1 | 1 | 1 | **7** | **Low** |
| **Özyörük, 2025** | 1 | 1 | 1 | 1 | 1 | 1 | 1 | **7** | **Low** |
| **Uren, 1987** | 0 | 0 | 0 | 0 | 0 | 1 | 0 | **1** | **High** |
| **Li, 2015** | 1 | 1 | 1 | 1 | 0 | 1 | 0 | **5** | **Moderate** |
| **Golender, 2024** | 1 | 1 | 1 | 1 | 1 | 1 | 1 | **7** | **Low** |
| **Mohapatra, 2022** | 1 | 1 | 1 | 1 | 1 | 1 | 0 | **6** | **Low** |
| **Bazargani, 2013** | 1 | 0 | 1 | 1 | 0 | 1 | 0 | **4** | **Moderate** |
| **Zahid, 2018** | 1 | 1 | 1 | 1 | 1 | 1 | 0 | **6** | **Low** |
| **Wenbin, 1991** | 0 | 0 | 0 | 0 | 0 | 1 | 0 | **1** | **High** |
| **Aziz-Boaron, 2013** | 1 | 1 | 1 | 1 | 0 | 1 | 0 | **5** | **Moderate** |
| **Chaisirirat, 2018** | 1 | 1 | 1 | 1 | 1 | 1 | 0 | **6** | **Low** |
| **Zheng, 2015** | 1 | 1 | 1 | 1 | 0 | 1 | 0 | **5** | **Moderate** |
| **Abu-Elzein, 2006** | 0 | 0 | 1 | 0 | 0 | 1 | 0 | **2** | **High** |
| **Abdullah, 2020** | 1 | 1 | 1 | 1 | 1 | 1 | 0 | **6** | **Low** |
| **Alkan, 2017** | 1 | 1 | 1 | 1 | 1 | 1 | 0 | **6** | **Low** |
| **Paksoy, 2024** | 1 | 1 | 1 | 1 | 1 | 1 | 1 | **7** | **Low** |
| **Yeruham, 2008** | 1 | 0 | 1 | 1 | 0 | 1 | 0 | **4** | **Moderate** |
| **Grobler, 2025** | 1 | 1 | 1 | 1 | 1 | 1 | 1 | **7** | **Low** |
| **Hwang, 2021** | 1 | 1 | 1 | 1 | 1 | 1 | 0 | **6** | **Low** |
| **Abayli, 2023** | 1 | 1 | 1 | 1 | 1 | 1 | 1 | **7** | **Low** |
| **Al-Sultany, 2013** | 1 | 0 | 1 | 1 | 0 | 1 | 0 | **4** | **Moderate** |
| **Finlaison, 2014** | 1 | 0 | 1 | 1 | 0 | 1 | 0 | **4** | **Moderate** |
| **Mirzaie, 2017** | 1 | 1 | 1 | 1 | 1 | 1 | 0 | **6** | **Low** |
| **Yeruham, 2003** | 1 | 0 | 1 | 1 | 0 | 1 | 0 | **4** | **Moderate** |
| **Farag, 1998** | 0 | 0 | 1 | 0 | 0 | 1 | 0 | **2** | **High** |
| **Behar, 2022** | 1 | 1 | 1 | 1 | 1 | 1 | 1 | **7** | **Low** |
| **Abu Elzein, 1999** | 0 | 0 | 1 | 0 | 0 | 1 | 0 | **2** | **High** |
| **Hijazeen, 2020** | 1 | 1 | 1 | 1 | 1 | 1 | 0 | **6** | **Low** |
| **Kim, 2015** | 1 | 1 | 1 | 1 | 0 | 1 | 0 | **5** | **Moderate** |
| **St. George, 1985** | 0 | 0 | 0 | 0 | 0 | 1 | 0 | **1** | **High** |
| **Dik, 2014** | 1 | 0 | 1 | 1 | 0 | 1 | 0 | **4** | **Moderate** |
| **Cybinski, 1983** | 0 | 0 | 0 | 0 | 0 | 1 | 0 | **1** | **High** |
| **Ogawa, 1992** | 0 | 0 | 1 | 0 | 0 | 1 | 0 | **2** | **High** |
| **Yeruham,2007** | 1 | 0 | 1 | 1 | 0 | 1 | 0 | **4** | **Moderate** |
| **Karayel-Hacioglu,2021** | 1 | 1 | 1 | 1 | 1 | 1 | 0 | **6** | **Low** |
| **Sah,2002** | 1 | 0 | 1 | 1 | 0 | 1 | 0 | **4** | **Moderate** |
| **Aziz-Boaron,2012** | 1 | 1 | 1 | 1 | 0 | 1 | 0 | **5** | **Moderate** |
| **Finlaison,2010** | 1 | 0 | 1 | 1 | 0 | 1 | 0 | **4** | **Moderate** |
| **Rezatofighi,2022** | 1 | 1 | 1 | 1 | 1 | 1 | 0 | **6** | **Low** |
| **Kun,2020** | 1 | 1 | 1 | 1 | 1 | 1 | 0 | **6** | **Low** |

**Supplementary Table 2. Country-specific posterior estimates of BEFV active infection prevalence from the Bayesian hierarchical model.**

| **Country** | **p_mean** | **p_median** | **p_ci_lower** | **p_ci_upper** |
| --- | --- | --- | --- | --- |
| **Australia** | 0.6368089 | 0.645226 | 0.313517 | 0.900639 |
| **China** | 0.3673234 | 0.355618 | 0.097016 | 0.719785 |
| **EGYPT** | 0.9135588 | 0.947197 | 0.646824 | 0.99879 |
| **Iran** | 0.0595687 | 0.050525 | 0.011661 | 0.169962 |
| **Iraq** | 0.221041 | 0.20197 | 0.056604 | 0.508561 |
| **Israel** | 0.3744149 | 0.366183 | 0.12498 | 0.685044 |
| **Pakistan** | 0.6699613 | 0.68168 | 0.354009 | 0.904765 |
| **Saudi Arabia** | 0.2329104 | 0.218731 | 0.060412 | 0.517319 |
| **South Africa** | 0.1659193 | 0.147752 | 0.035516 | 0.414245 |
| **Taiwan** | 0.9970375 | 0.998259 | 0.98699 | 0.999905 |
| **Thailand** | 0.2993229 | 0.281189 | 0.079743 | 0.627891 |
| **Turkey** | 0.8120237 | 0.833381 | 0.467029 | 0.998147 |

**Note**:This table presents posterior estimates of true BEFV active infection prevalence for each country included in the meta-analysis, derived from the misclassification-adjusted Bayesian hierarchical model. For each country, the table reports the posterior mean prevalence, the median, and the 95% credible interval (CrI) representing the uncertainty propagated from sampling error as well as from assay sensitivity and specificity.

**Supplementary Table 3. Country-level posterior estimates of true BEFV seroprevalence from the Bayesian hierarchical model.**

| **Country** | **p_mean** | **p_median** | **p_ci_lower** | **p_ci_upper** |
| --- | --- | --- | --- | --- |
| **Australia** | 0.118084 | 0.108214 | 0.036808 | 0.257518 |
| **China** | 0.352074 | 0.345075 | 0.147906 | 0.597895 |
| **EGYPT** | 0.470464 | 0.468776 | 0.139847 | 0.80618 |
| **India** | 0.311313 | 0.300456 | 0.116046 | 0.569583 |
| **Israel** | 0.1534 | 0.142212 | 0.051119 | 0.320536 |
| **Jordan** | 0.380805 | 0.375911 | 0.163678 | 0.633899 |
| **Pakistan** | 0.843629 | 0.856102 | 0.652131 | 0.960333 |
| **Saudi Arabia** | 0.116238 | 0.106515 | 0.037186 | 0.252373 |
| **South Korea** | 0.0725 | 0.065283 | 0.021819 | 0.165894 |
| **Turkey** | 0.838169 | 0.857694 | 0.591398 | 0.973776 |
| **Zimbabwe** | 0.338444 | 0.32206 | 0.132665 | 0.644134 |

**Note**:Posterior summaries of country-specific true seroprevalence (p) estimated under the Bayesian hierarchical misclassification model. For each country, *p_mean* represents the posterior mean estimate of true seroprevalence, *p_median* is the posterior median, and *p_ci_lower* and *p_ci_upper* denote the bounds of the 95% Bayesian credible interval (CrI), corresponding to the 2.5th and 97.5th percentiles of the posterior distribution. These estimates reflect adjustment for imperfect diagnostic test accuracy and partial pooling across studies, allowing comparisons across heterogeneous surveillance systems and sampling periods

**Supplementary Table 4. Clinical signs reported or inferred for BEF clinical-diagnosis studies**

| **Study (Author, Year)** | **Clinical signs** |
| --- | --- |
| **Uren, 1987** | Fever, lameness, recumbency (inability to rise), agalactia (cessation of milk production); mortality was also noted |
| **Ogawa, 1992** | Sudden fever up to 41–42 °C, anorexia, salivation, cutaneous muscle tremor, lameness, and instability when standing |
| **Liao, 1998** | Pyrexia (high fever), excessive nasal discharges, labored respiration/dyspnea, salivation, lameness, depression, reduced milk yield |
| **Wang, 2001** | Sudden onset of fever, stiffness, lameness, nasal and ocular discharges, depression, cessation of rumination, and constipation |
| **Sah, 2002** | Sudden fever (105 °F), loss of appetite, lameness/stiff gait, sharp fall in milk production, constipation or diarrhoea, laboured respiration, lacrimation, nasal discharge, drooling saliva, cough, muscular stiffness, recumbency, rumen atony |
| **Yeruham, 2003** | Fever, depression, stiffness, lameness, recumbency, decreased milk yield, and transient paralysis in severe cases |
| **Hsieh, 2005** | Sudden fever, depression, stiffness, lameness, nasal and ocular discharge, reduced appetite, cessation of rumination, and decreased milk production |
| **Abu-Elzein, 2006** | Fever, depression, stiffness, lameness, nasal discharge, salivation, reduced milk production, and recumbency |
| **Yeruham, 2007** | Acute fever, depression, stiffness, lameness, reluctance to move, recumbency, decreased feed intake, and transient drop in milk production |
| **Yeruham, 2008** | Sudden fever, depression, muscle stiffness, lameness, reluctance to move, recumbency, and marked decrease in milk production |
| **Aziz-Boaron, 2012** | Acute fever, depression, stiffness, lameness, reluctance to move, mild respiratory distress, and transient reduction in milk yield |
| **Bazargani, 2013** | Fever, hyperpnoea, mouth breathing, subcutaneous emphysema, pneumoperitoneum, anorexia, sticky nasal discharge, hypersalivation, and depression |
| **Islam, 2018** | High fever, swelling of pre-scapular and pre-femoral lymph nodes, weakness, inappetence, lacrimation, pale conjunctival mucosae, increased respiration and pulse rate, decreased milk yield, and loss of body condition |
| **Özyörük, 2025** | Rapid onset, biphasic fever, lethargy, anorexia, cessation of lactation, unsteady gait, respiratory distress, muscle tremors, and rapid recovery |

**Supplementary Figure 1.** Forest plot of BEFV prevalence by PCR.

**----------------------------------------------------------------------------------------------------------------------------------**

**Supplementary Figure 2.** Forest plot of BEFV prevalence by ELISA.

**----------------------------------------------------------------------------------------------------------------------------------**

**Supplementary Figure 3.** Forest plot of BEFV prevalence by Virus Neutralization Test (VNT).

**----------------------------------------------------------------------------------------------------------------------------------**

**Supplementary Figure 4.** Forest plot of BEFV prevalence by Virus Isolation.

**----------------------------------------------------------------------------------------------------------------------------------**

**Supplementary Figure 5.** Forest plot of BEFV prevalence in Bos taurus (25 studies).

**----------------------------------------------------------------------------------------------------------------------------------**

**Supplementary Figure 6.** Forest plot of BEFV prevalence in Bubalus bubalis (10 studies).

**----------------------------------------------------------------------------------------------------------------------------------**

**Supplementary Figure 7.** Forest plot of BEFV prevalence in Bos grunniens (2 studies).

**----------------------------------------------------------------------------------------------------------------------------------**

**Supplementary Figure 8.** Forest plot of BEFV prevalence in Capra hircus (3 studies).

**----------------------------------------------------------------------------------------------------------------------------------**

**Supplementary Figure 9.** Forest plot of BEFV prevalence in Ovis aries (3 studies).

**----------------------------------------------------------------------------------------------------------------------------------**
